# Supplementary material for: Effects of an integrated mobile health lifestyle intervention among overweight and obese women planning for pregnancy in Singapore: protocol for the single-arm healthy early life moments in Singapore (HELMS) study
Source: BMJ Open. 2022 Dec 12;12(12):e061556. doi: 10.1136/bmjopen-2022-061556 (PMC9748919; doi:10.1136/bmjopen-2022-061556)
Supplement: Supplementary data [file bmjopen-2022-061556supp001.pdf]

Restricted, Sensitive (Normal)

SingHealth

PARTICIPANT INFORMATION SHEET AND CONSENT FORM

You are being invited to participate in a research study. Your participation in this study is entirely voluntary. Before you take part in this research study, the study must be explained to you and you must be given the chance to ask questions. Your questions will be answered clearly and to your satisfaction. Please read carefully the information provided here. If you agree to participate, please sign the consent form. You will be given a copy of this document.

If you are a parent or legal guardian giving consent for a child to participate in the study, please note that the word "you" refers to your child.

STUDY INFORMATION

Protocol Title:
Healthy Early Life Moments in Singapore (HELMS)

Principal Investigator:
Prof. Jerry Chan Kok Yen
Senior Consultant
Department of Reproductive Medicine
KK Women's and Children's Hospital
100 Bukit Timah Road
Singapore 229899

PURPOSE OF THE RESEARCH STUDY

The purpose of this study is to implement a behavioural intervention to address the challenges of poor outcomes relating to fertility, perinatal and eventual chronic diseases through an integrated continuum of care from the preconception phase, across maternity until the early postnatal phase. We hope to learn the optimal life course model of care in breaking vicious life cycles and promote virtuous life cycles for our women, children and family.

You were selected as a possible participant in this study because you are planning for a child in the near future and falling within the body mass index (BMI) of 25 to 40 kg/m^2.

This study targets to recruit 300 participants from the general population in Singapore where the study visits will take place at KK Women's and Children's Hospital.

STUDY PROCEDURES & YOUR RESPONSIBILITIES IN THIS STUDY

If you agree to take part in this study, you will be asked to begin participating before you are pregnant, to attend the study visits during pregnancy and for a further 18 months after the birth of your child.

Chan JKY, *et al. BMJ Open* 2022; 12:e061556. doi: 10.1136/bmjopen-2022-061556

Restricted, Sensitive (Normal)

The table below indicates the study visits and the procedures which will be done at each visit:

Study visit / Procedure	Preconception					Pregnancy		Delivery	Postnatal					
	Base-line	3m	6m	9m	Exit/ 6-10w	24-28w	32-36w	Birth	1-2w	6-8w	4m	6m	12m	18m
Questionnaires	✓	Phone calls	✓	Phone calls	✓	✓	✓		✓	✓	✓	✓	✓	✓
Anthropometry	✓		✓		✓	✓	✓		✓	✓	✓	✓	✓	✓
Eye assessments	✓		✓		✓		✓							
Blood collection	✓ (35ml)		✓ (10ml)		✓ (25ml)	✓ (30ml)		✓ (10ml + cord blood 20ml)		✓ (30ml)				✓ (30ml)
Placenta collection								✓						
Stool collection	✓	✓	✓	✓	✓	✓	✓	✓ (C)	✓ (C)	✓ (C&M)	✓ (C)	✓ (C)	✓ (C)	✓ (C)
Breast milk collection									✓	✓	✓	✓	✓	
Activity tracker	✓		✓			✓				✓			✓	
Sleep tracker	✓ (spanning all visits)													

*C: child, M: mother

You will be provided with lifestyle intervention initiatives since preconception (before pregnancy), throughout pregnancy and until 18 months postnatal (after delivery of baby). Throughout the study, you will be

- asked to administer a nutrition intervention tool known as '6P' to assess and monitor your dietary behaviour;
- prescribed with multi-micronutrients supplements by the study team doctors;
- provided with phase specific education materials related to preconception care, pregnancy care and dyad care;
- provided with exercise guidance;
- provided with Vitamin D drops for your child.

You will need to visit the study clinics for

- 3 times during the preconception phase (2 times if successfully getting pregnant over 1 year after enrollment into the study);
- 3 times during the pregnancy phase; and
- 6 times during the postnatal phase.

Each visit will take approximately 2 hours to complete. Additional visits may be required depending on the clinical care investigations and reviews as per necessary. A feedback survey will be performed at the end of each preconception, pregnancy and postnatal phases and upon study exit for women who are withdrawn at specific phase of the study.

Details of study measures at respective time-point are described below:

Preconception:
At the first visit, your will be asked to come after a minimum of 8 hours overnight fasting. You will be

- screened for eligibility;
- assessed for lifestyles, sexual health and anthropometry (weight, height, waist-hip circumference);
- undergoing an eye assessment including retinal photography to take a photo at the back of your eye.
- undergoing blood collection, at most 35ml of blood (less than 3 tablespoons):
    - Blood lipids (cholesterol and triglycerides)

Chan JKY, *et al. BMJ Open* 2022; 12:e061556. doi: 10.1136/bmjopen-2022-061556

- Hormonal profile
- Infection screen
- Full blood count (FBC)
- Oral glucose tolerance test (OGTT)*
- Insulin (hormone which regulates blood glucose levels)
- C-peptide (a marker of insulin production)
- HbA1c (blood test that gives an indication of your average blood glucose levels over the past three months)
- Additional blood for research analyses (12ml / less than 1 tablespoon)

- self-administering a nutrition intervention tool, known as the 6P tool to assess and monitor your dietary behaviour
- receiving education related to preconception health from the clinical staff/ doctors
- receiving exercise guidance and an activity tracker (Actigraph device, Figure 1) to track for your 24-hour activity. The tracker is to be worn on the wrist and captures activity levels, sleep and light exposure. Arrangement will be made to collect the tracker back from you after 10 days.

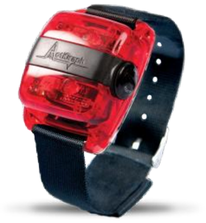

Figure 1: Actigraph device

* A 75g (approximately 5 tablespoons) oral glucose tolerance test (OGTT) to determine how quickly sugar is cleared from your blood. This involves a total of 2 blood-taking time points; at fasting and 2 hours after drinking the glucose drink. At the 2-hour time point, we will also collect blood for triglycerides, insulin and C-peptide (approximately 5ml / 1 teaspoon). Your blood will be taken from your arm at each time-point.

We will also make arrangement to collect stool sample from your home. The stool sample will be used for research analyses.

You will receive preconception care provided by the doctors and follow-up visits will be arranged based on the clinical reviews/investigations as per necessary. Blood test results will be reviewed by the doctors.

6 months after the first visit, you will be followed up in the clinic for 6P, personality, lifestyles, physical activity and eye assessments, health and weight review as per necessary. 10ml (2 teaspoons) of blood will be collected for fasting glucose, lipid profile and insulin tests. Stool sample will also be collected at this time point.

At 3, 9 and 12 months, you will be followed-up through phone calls to track for pregnancy status and asked to self-administer the 6P tool for dietary behaviour monitoring and to continue with the exercises. Arrangement will also be made to collect the stool samples from your home. Throughout the preconception phase, preconception health and dietary related messages will be delivered to you by the study team.

During the preconception follow-up, if you have a positive urinary pregnancy test, an ultrasound scan will be arranged for you to confirm clinical pregnancy. If you do not conceive within one year from the first visit, you will be contacted to arrange for a final anthropometry, lifestyles, eye assessments and blood tests (FBC, fasting glucose, insulin, C-peptide, HbA1c,

lipid profile and some additional blood for research analyses) before withdrawal from the study. Another stool sample will be collected as well.

Pregnancy:
There will be 3 visits (6-10, 24-28 and 32-36 weeks) during your pregnancy that coincide with your routine antenatal visits, where lifestyle and anthropometry assessments will be performed. The 6P tool will also be administered at each visit and blood test reviews will be provided by the doctor.

By tagging along with routine antenatal clinical blood draw, we will collect additional blood at the same time:

- 6-10 weeks: Lipid profile, FBC, fasting glucose, insulin, C-peptide, HbA1c and additional blood for research analyses.
- 24-28 weeks: Lipid profile, FBC, OGTT, insulin, C-peptide and additional blood for research analyses.

As per standard clinical practice, the OGTT during pregnancy consists of 3 time points: fasting, 1 and 2 hour(s) after drinking the glucose drink. We will also collect blood for triglycerides, insulin and C-peptide (approximately 5ml / 1 teaspoon) at the 1 and 2-hour time-points.

In total, at most 30ml of blood (2 tablespoons) will be collected at each visit, inclusive of 12ml (less than 1 tablespoon) for research analyses mentioned above.

At 24-28 weeks, you will receive an activity tracker to track for your 24-hour activity. Arrangement will be made to collect the tracker back from you after 10 days.

At 6-10 and 32-36 weeks, eye assessment will be done.

Stool samples will be collected from you for the 3 visits, arrangement will be made to collect the stool samples from your home. The stool samples will be used for research analyses.

You will also receive trimester-specific education on antenatal care, healthy lifestyles and exercise guidance throughout the pregnancy phase.

Delivery:
After you have delivered, placenta and 20 ml (less than 2 tablespoons) of cord blood will be collected if you have given consent. It is likely that there will not be enough cord blood left for banking after it has been collected for study purposes. However, if you wish to bank your child's cord blood at any point during the study, your decision will be respected by the study team. 10ml (2 teaspoons) of your blood will be collected and stool sample from your child will also be collected for research analyses.

Postnatal:
After delivery, there will be 6 visits (1-2 weeks, 6-8 weeks, 4, 6, 12 and 18 months) during the postnatal phase coinciding with the child vaccination time points. During these visits, there will be

- lifestyle and anthropometry assessments;
- dyad care by the clinical staff/ doctors for both mother and child, including reviews on dietary measures and weight outcomes/ child growth monitoring;
- continuous intervention emphasising on dietary behaviours, physical activity and lifestyle changes

At 6-8 weeks and 18 months, OGTT, insulin, C-peptide, HbA1c and lipid profile assessment will be performed. In total, at most 30ml of blood (2 tablespoons), inclusive of 12ml (less than 1 tablespoon) for research analyses, will be collected at the respective visits.

At 6-8 weeks and 12 months, you will also receive an activity tracker to track for your 24-hour activity. Arrangement will be made to collect the tracker back from you after 10 days.

We would also like to collect 10ml (2 teaspoons) of breast milk up to the 12-month visit or until the cessation of lactation, whichever is earlier. Stool samples will be collected from your child for the 6 visits and from you at 6-8 weeks, arrangement will be made to collect the stool samples from your home. The breast milk and stool samples will be used for research analyses.

The human biological material may be tested in Singapore or Norway, and only coded human biological materials and/or data will be transferred out of Singapore. To protect your confidentiality, all the human biological materials will be coded. All identifiable information (e.g., names, IC numbers) will be kept separate from the human biological materials. The link between your identifiable information and the code number will be kept confidential by the Principal Investigator or a trusted third-party. The human biological materials and data collected will not be used in research involving human-animal combinations, which is restricted by laws imposed by the Ministry of Health, Singapore.

Sleep tracking
You will receive a device (Oura Ring, Figure 2) to track your sleeping patterns throughout the study period. The device can be worn on any finger (except the thumb) and records movement, heart rate and temperature. The device is water-proof up to 100m and can be worn while showering or swimming. Wearing the device while scuba diving or keeping it submerged underwater for over 12 hours should be avoided. Arrangement will be made to collect the device back from you upon study completion.

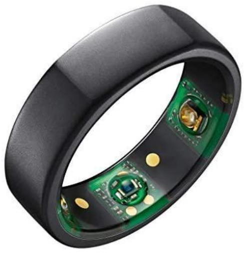

Figure 2: Oura Ring

If you agree to participate in this study, you should follow the study visit schedules, measures, advice and directions given to you by the study team.

WHAT IS NOT STANDARD CARE OR IS EXPERIMENTAL IN THIS STUDY

The study is being conducted because the lifestyle intervention designed to start from the preconception, through pregnancy until the postnatal phase are not yet proven to be a standard care in women with high BMI. We hope that your participation will help us to determine whether the intervention is equal or superior to existing clinical care.

Although anthropometric measurement and retinal photography, as well as blood glucose, FBC and lipid profile tests may be part of standard medical care, in this study these procedures are being performed for the purposes of the research, and are not part of your routine care.

POSSIBLE RISKS, DISCOMFORTS OR INCONVENIENCES

Personal privacy and confidentiality:
This study uses health information that may affect your privacy. To protect your confidentiality, only a unique code number will be used to identify data and/or biological material that we collected from you.

As there will be a link between the code and your identifiable information, there is still a possibility of data breach. A data breach is when someone sees or uses data without permission. If there is a data breach, someone could see or use the data we have about you. Even without your name, there is a chance someone could figure out who you are. They could misuse your data. We believe the chance of this is very small, but it is not zero.

Questionnaires/ surveys/ interviews:
Some of the questions might make you feel uncomfortable or upset. You may refuse to answer any of the questions and/or take a break at any time during the study.

Collection of blood:
Taking blood may cause momentary discomfort, pain, bleeding, bruising or swelling at the site of the needle stick. Rarely, taking blood may cause fainting or infection. If possible, the research blood sample(s) will be collected at the same time you have blood drawn for clinical care or through an existing catheter already inserted into a vein.

Collection of placenta:
Collection of placenta is safe, non-invasive and there will be no adverse effects.

Collection of stool and breast milk samples:
Collection of stool and breast milk may cause inconveniences and momentary discomfort.

Retinal photography:
Retinal photography may cause mild and temporary discomfort. You may experience the flashlight as very intense and see spots for a short time following the examination.

Oura Ring:
Wearing the device may cause mild and temporary discomfort. If you experience redness or skin irritation on your finger, remove the ring immediately.

POTENTIAL BENEFITS

If you participate in this study, you may reasonably expect to benefit from the study by receiving lifestyle intervention and relevant education starting from preconception, through pregnancy until the postnatal phase.

ALTERNATIVE PROCEDURES/ TREATMENTS IF YOU DO NOT PARTICIPATE IN THE STUDY

If you choose not to take part in this study, the alternative is to have what is considered standard care for your condition. In our institution, this would be routine clinical care for women

Restricted, Sensitive (Normal)

at who are trying for pregnancy, during and after pregnancy, and you do not need to undergo the research activities mentioned above.

COSTS & PAYMENTS IF PARTICIPATING IN THIS STUDY

There is no cost to you for participating in this research study.

If you take part in this study, the following will be performed at no charge to you:

- All assessments and examinations done at the HELMS study clinic
- Preconception hormonal blood tests, infection screen, OGTT, fasting glucose, insulin, C-peptide, HbA1c, lipid profile and FBC at the HELMS study visits
- Multi-micronutrients supplements
- Vitamin D drops for your child
- Doctor consultations during the preconception phase and 6-10w visit during pregnancy phase at the HELMS study clinic

These costs will be borne by KK Women's and Children's Hospital.

The cost of your usual medical care (procedures, medications and doctor visits) will continue to be billed to you.

You will be reimbursed for your time, inconvenience and transportation costs. Reimbursement for each study specific visit will be as follows:

- Preconception phase:
  a. S$70 for baseline visit
  b. S$60 for 6m visit
  c. S$100 for 12m/Exit visit
- Pregnancy phase:
  a. S$100 for 6-10w visit
  b. S$100 for 24-28w visit
  c. S$80 for 32-36w visit
- Delivery: S$20 for placenta, maternal and cord blood collection
- Postnatal phase:
  a. S$100 for 1-2w visit
  b. S$100 for 6-8w visit
  c. S$80 for 4m, 6m and 12m visit
  d. S$120 for 18m visit
- Reimbursement upon submission:
  a. S$30 for participation in Actigraph data collection where the device is worn for 10 consecutive days
  b. S$50 for participation in Oura Ring data collection where the device is worn for at least 3 consecutive months in each phase
  c. S$5 for each stool collection.
  d. S$10 for each breast milk collection.

Chan JKY, *et al. BMJ Open* 2022; 12:e061556. doi: 10.1136/bmjopen-2022-061556

Restricted, Sensitive (Normal)

INCIDENTAL FINDINGS

There will not be any incidental findings arising in this research. “Incidental findings” are findings that have potential health or reproductive importance to research participants like you and are discovered in the course of conducting the study, but are unrelated to the purposes, objectives or variables of the study.

WHAT HAPPENS TO THE SAMPLES COLLECTED FOR THE RESEARCH

The biological materials collected for this research study will be deemed to be donated to KK Women’s and Children’s Hospital as a gift. By agreeing to this, you give up your rights to the biological materials. If the use of your biological materials and/or your data results in intellectual property rights and commercial benefits, you will not receive any financial benefits or proprietary interest.

The biological materials will be used only for the purpose of this research and will be discarded or destroyed upon completion of the research study.

PARTICIPANT’S RIGHTS

Your participation in this study is entirely voluntary. You have a right to ask questions, which the study team will do their best to answer clearly and to your satisfaction.

In the event of any new information becoming available that may be relevant to your willingness to continue in this study, you (or your legal representative, if relevant) will be informed in a timely manner by the Principal Investigator or his/her representative and will be contacted for further consent if required.

WITHDRAWAL FROM STUDY

You are free to withdraw your consent and discontinue your participation in the study at any time, without your medical care being affected. If you decide to stop taking part in this study, you should tell the Principal Investigator.

However, any of your data and your child’s data that have been collected until the time of your withdrawal will be kept and analysed. Your or your child’s medical information will be retrieved from the hospital medical records even after your withdrawal. The reason is to enable a complete and comprehensive evaluation of the study.

The biological materials that have been collected for the study will not be returned to you. However, you retain your right to ask the Principal Investigator to discard or destroy any remaining samples if they have not been anonymised and/or have not been used.

Your study doctor, the Principal Investigator of this study may stop your participation in the study at any time for one or more of the following reasons:

- Failure to follow the instructions of the Principal Investigator and/or study staff.
- The Principal Investigator decides that continuing your participation could be harmful to your health or safety.
- You require treatment not allowed in the study.
- The study is cancelled.

Chan JKY, *et al. BMJ Open* 2022; 12:e061556. doi: 10.1136/bmjopen-2022-061556

RESEARCH RELATED INJURY AND COMPENSATION

If you follow the directions of the Principal Investigator of this research study and you are injured due to the research procedure(s) given under the plan for the research study, our institution will provide you with the appropriate medical treatment.

Payment for management of the normally expected consequences of your treatment (i.e. consequences of your treatment which are not caused by your participation in the research study) will not be provided.

You still have all your legal rights. Nothing said here about treatment or compensation in any way alters your right to recover damages where you can prove negligence.

CONFIDENTIALITY OF STUDY AND MEDICAL RECORDS

Your participation in this study will involve the collection of Personal Data. “Personal Data” means data about you which makes you identifiable (i) from such data or (ii) from that data and other information which an organisation has or likely to have access. Examples of personal data include name, national registration identity card (NRIC), nationality, passport information, date of birth, and telephone number.

Personal Data collected for this study will be kept confidential. Your study records and medical records, to the extent required by the applicable laws and regulations, will not be made publicly available. Only the study team will have access to the personal data being collected from you. In the event of any publication regarding this study, your identity will remain confidential.

However, the monitor(s), the auditor(s), the Institutional Review Board, and the regulatory authority(ies) will be granted direct access to your original medical records and study records to verify study procedures and data, without making any of your information public. The HELMS study may gain access to your/ your child’s information records held by healthcare providers and government agencies (e.g. Singapore’s National Disease Registries, National Immunisation Registry, Ministry of Health, Ministry of Education) in Singapore for the purpose of studying health outcomes.

By signing the Consent Form, you consent to (i) the collection, access to, use and storage of your Personal Data by KK Women’s and Children’s Hospital, and (ii) the disclosure of such Personal Data to our authorised service providers and relevant third parties as mentioned above.

Any information containing your Personal Data that is collected for the purposes of this research will be stored in Singapore. To protect your identity, your Personal Data will be labelled with a unique code number. The code will be used in place of your name and other information that directly and easily identifies you. The study team will keep a separate file that links your code number to your Personal Data. This will be kept in a safe place with restricted access. In the event of any international collaboration in data analysis, your coded data will be transferred out of Singapore.

All data collected in this study are the property of KK Women’s and Children’s Hospital. The data will be used for the purpose of this research study only, unless you give permission for

your data to be made available for future use in other research studies. For this purpose, consent for future research will be sought from you.

By participating in this research study, you are confirming that you have read, understood and consent to the SingHealth Data Protection Policy, the full version of which is available at www.singhealth.com.sg/pdpa.

WHO HAS REVIEWED THE STUDY

This study has been reviewed by the SingHealth Centralised Institutional Review Board for ethics approval.

If you have questions about your rights as a participant, you can call the SingHealth Centralised Institutional Review Board at 6323 7515 during office hours (8:30 am to 5:30pm).

WHO TO CONTACT IF YOU HAVE QUESTIONS REGARDING THE STUDY

If you have questions about this research study or in the case of any injuries during the course of this study, you may contact:

Principal Investigator
Prof. Jerry Chan Kok Yen
Department of Reproductive Medicine
KK Women's and Children's Hospital
100 Bukit Timah Road
Singapore 229899
Tel: 6394 1060 / 8125 3639 (after office hours)

If you have any feedback about this research study, you may contact the Principal Investigator or the SingHealth Centralised Institutional Review Board.

Restricted, Sensitive (Normal)

CONSENT FORM FOR RESEARCH STUDY

Protocol Title:
Healthy Early Life Moments in Singapore (HELMS)

Principal Investigator:
Prof. Jerry Chan Kok Yen
Department of Reproductive Medicine
KK Women's and Children's Hospital
100 Bukit Timah Road
Singapore 229899

I agree to participate in the research study as described and on the terms set out in the Participant Information Sheet.

The nature, risks and benefits of the study have been explained clearly to me and I fully understand them.

I understand the purpose and procedures of this study. I have been given the Participant Information Sheet and the opportunity to discuss and ask questions about this study and am satisfied with the information provided to me.

I understand that my participation is voluntary and that I am free to withdraw at any time, without giving any reasons and without my medical care being affected.

By participating in this research study, I confirm that I have read, understood and consent to the SingHealth Data Protection Policy.

Should withdrawal from the study occur, please indicate your choice using the relevant checkbox.

☐ I do not agree to be contacted for other related research studies after withdrawal.
☐ I agree to be contacted for other related research studies after withdrawal.

Name of participant	Signature/Thumbprint (Right / Left)	Date of signing

Chan JKY, *et al. BMJ Open* 2022; 12:e061556. doi: 10.1136/bmjopen-2022-061556

Restricted, Sensitive (Normal)

To be completed by parent / legal guardian / legal representative, where applicable

I hereby give consent for _________________________ (Name of Participant) to participate in the research study. The nature, risks and benefits of the study have been explained clearly to me and I fully understand them.

I confirm that I have read, understood and consent to the SingHealth Data Protection Policy.

____________________	____________________________	______________
Name of participant's parent/ legal guardian/ legal representative	Signature/Thumbprint (Right / Left)	Date of signing

To be completed by translator, if required

The study has been explained to the participant/ legal representative in

________________________________ by _____________________________________.

Language Name of translator

To be completed by witness, where applicable

I, the undersigned, certify that:

- I am 21 years of age or older.
- To the best of my knowledge, the participant or the participant's legal representative signing this informed consent form had the study fully explained to him/her in a language understood by him/ her and clearly understands the nature, risks and benefits of the participant's participation in the study.
- I have taken reasonable steps to ascertain the identity of the participant or the participant's legal representative giving the consent.
- I have taken reasonable steps to ascertain that the consent has been given voluntarily without any coercion or intimidation.

Witnessed by: ________________________________ ___________________

Name of witness Date of signing

____

Signature of witness

1. An impartial witness (who is 21 years of age or older, has mental capacity, who is independent of the research study, and cannot be unfairly influenced by people involved with the research study) should be present during the entire informed consent discussion if a participant or the participant's legal representative is unable to read, and/or sign and date on the consent form (i.e. using the participant's or legal representative's thumbprint). After the written consent form and any written information to be provided to participant is read and explained to the participant or the participant's legal representative, and after the participant or the participant's legal representative has orally consented to the participant's participation in the study and, if capable of doing so, has signed and personally dated the consent form, the witness should sign and personally date the consent form. This is applicable for Clinical Trials regulated by HSA and Human Biomedical Research under the HBRA.

2. For HBRA studies, the witness may be a member of the team carrying out the research only if a participant or the participant's legal representative is able to read, sign and date on the consent form.

Chan JKY, *et al. BMJ Open* 2022; 12:e061556. doi: 10.1136/bmjopen-2022-061556

Restricted, Sensitive (Normal)

Investigator's Statement

I, the undersigned, certify to the best of my knowledge that the participant/ participant's legal representative signing this consent form had the study fully explained to him/her and clearly understands the nature, risks and benefits of the participant's participation in the study.

Name of Investigator/ Person obtaining consent	Signature	Date

Chan JKY, *et al. BMJ Open* 2022; 12:e061556. doi: 10.1136/bmjopen-2022-061556

Restricted, Sensitive (Normal)

INFORMATION & CONSENT FORM FOR FUTURE RESEARCH

This is an optional component that is separate from the research study. You may still participate in the research study if you say "No" to this. Please ask questions if you do not understand why we are asking for your permission.

In this Consent Form for Future Research, we seek your permission to keep your data for future research. The data will be kept in KK Women's and Children's Hospital. Except if you withdraw your consent or there are limits imposed by law, there is no limit on the length of time we will store your data. Researchers will use your data for research long into the future.

This is what will be done with your stored data:

- We may use the data to answer additional research questions in other research studies. This is outside the scope of the research study but still related to maternal-child health and disease development.
- We may share the data with other researchers at Agency for Science, Technology and Research (A*STAR), National University of Singapore, Duke-NUS Medical School, Singapore Management University etc. and with researchers outside of Singapore, such as collaborators from University of Southampton, UK etc.
- The stored data will be labelled with a code instead of information that directly identifies you (e.g. your name, NRIC, date of birth, etc.). We will keep a separate file (key) that links your code to your identifiable information.
- When we share your data with other researchers, it will be in a coded manner. They will not be able to identify you from the coded data.
- If you decide at a later time that you do not want your data to be used for future research, you can contact the Principal Investigator or study team at any time. All your stored data that has not been used or shared with other researchers will be removed and discontinued from further use, unless this information is already included in analyses or used in publications.

Chan JKY, *et al. BMJ Open* 2022; 12:e061556. doi: 10.1136/bmjopen-2022-061556

Restricted, Sensitive (Normal)

CONSENT FORM FOR FUTURE RESEARCH

This component is optional. You do not have to agree to it in order to participate in the research study.

Please indicate your choice using the relevant checkbox.

☐ I do not agree to have my data stored for future use in other research studies.
☐ I agree to have my data stored for future use in other research studies.

Name of participant	Signature/Thumbprint (Right / Left)	Date of signing

To be completed by parent / legal guardian / legal representative, where applicable

I hereby give consent for ________________________ (Name of Participant)'s data obtained from the research study to be stored for future use in other research studies in the interest of medical progress as described in and on terms set out in the Information & Consent Form for Future Research.

I understand that his/her participation is voluntary and I can withdraw his/her participation at any time, without giving reasons.

The nature of this optional component has been explained clearly to me and I fully understand them.

I have been given the Information & Consent Form for Future Research and the opportunity to discuss and ask questions about this optional component and am satisfied with the information provided to me.

I confirm that I have read, understood and consent to the SingHealth Data Protection Policy.

Name of participant's parent/ legal guardian/ legal representative	Signature/Thumbprint (Right / Left)	Date of signing

To be completed by translator, if required

The optional component (storage of data for future use in other research studies) has been explained to the participant/ participant's legal representative in

________________________________ by ___________________________________.
Language Name of translator

Restricted, Sensitive (Normal)

To be completed by witness, where applicable

I, the undersigned, certify that:

- I am 21 years of age or older.
- To the best of my knowledge, the participant or the participant's legal representative signing this Information & Consent Form for Future Research had the optional component fully explained to him/her in a language understood by him/ her and clearly understands the purpose and the nature of the participant's participation in the study.
- I have taken reasonable steps to ascertain the identity of the participant or the participant's legal representative giving the consent.
- I have taken reasonable steps to ascertain that the consent has been given voluntarily without any coercion or intimidation.

Witnessed by: ________________________________ ____________________

Name of witness Date of signing

____

Signature of witness

1. An impartial witness (who is 21 years of age or older, has mental capacity, who is independent of the research study, and cannot be unfairly influenced by people involved with the research study) should be present during the entire informed consent discussion if a participant or the participant's legal representative is unable to read, and/or sign and date on the consent form (i.e. using the participant's or legal representative's thumbprint). After the written consent form and any written information to be provided to participant, is read and explained to the participant or the participant's legal representative, and after the participant or the participant's legal representative has orally consented to the participant's participation in the study and, if capable of doing so, has signed and personally dated the consent form, the witness should sign and personally date the consent form. This is applicable for Clinical Trials regulated by HSA and Human Biomedical Research under the HBRA.

2. For HBRA studies, the witness may be a member of the team carrying out the research only if a participant or the participant's legal representative is able to read, sign and date on the consent form.

Investigator's Statement

I, the undersigned, certify to the best of my knowledge that the participant/ participant's legal representative signing this Information & Consent Form for Future Research had the optional component (storage of data for future use in other research studies) fully explained to him/her and clearly understands the purpose and the nature of the participant's participation in the study.

________________________ ________________________ ________________

Name of Investigator/ Person obtaining consent | Signature | Date

Chan JKY, *et al. BMJ Open* 2022; 12:e061556. doi: 10.1136/bmjopen-2022-061556
