# Supplementary material for: Effects of an integrated mobile health lifestyle intervention among overweight and obese women planning for pregnancy in Singapore: protocol for the single-arm healthy early life moments in Singapore (HELMS) study
Source: BMJ Open. 2022 Dec 12;12(12):e061556. doi: 10.1136/bmjopen-2022-061556 (PMC9748919; doi:10.1136/bmjopen-2022-061556)
Supplement: Author's manuscript [file bmjopen-2022-061556.draft_revisions.pdf]

BMJ Open is committed to open peer review. As part of this commitment we make the peer review history of every article we publish publicly available.

When an article is published we post the peer reviewers' comments and the authors' responses online. We also post the versions of the paper that were used during peer review. These are the versions that the peer review comments apply to.

The versions of the paper that follow are the versions that were submitted during the peer review process. They are not the versions of record or the final published versions. They should not be cited or distributed as the published version of this manuscript.

BMJ Open is an open access journal and the full, final, typeset and author-corrected version of record of the manuscript is available on our site with no access controls, subscription charges or pay-per-view fees (http://bmjopen.bmj.com).

If you have any questions on BMJ Open's open peer review process please email info.bmjopen@bmj.com

BMJ Open

# Healthy Early Life Moments in Singapore (HELMS): study protocol for a single-arm pilot implementation model



| | |
|---|---|
| Journal: | *BMJ Open* |
| Manuscript ID | bmjopen-2022-061556 |
| Article Type: | Protocol |
| Date Submitted by the Author: | 29-Jan-2022 |





| | |
|---|---|
| Complete List of Authors: | Chan, Jerry; KK Women's and Children's Hospital, Department of Reproductive Medicine; Duke-NUS Medical School<br>Ku, Chee Wai; KK Women's and Children's Hospital, Department of Reproductive Medicine; Duke-NUS Medical School,<br>Loy, See Ling; KK Women's and Children's Hospital, Department of Reproductive Medicine; Duke-NUS Medical School<br>Godfrey, Keith; University of Southampton, MRC Lifecourse Epidemiology Unit; University of Southampton, National Institute for Health Research Southampton Biomedical Research Centre<br>Fan, Yiping; KK Women's and Children's Hospital, Department of Reproductive Medicine<br>Chua, Mei Chien; Duke-NUS Medical School; KK Women's and Children's Hospital, Department of Neonatology<br>Yap, Fabian; KK Women's and Children's Hospital, Department of Paediatrics; NUS Yong Loo Lin School of Medicine |





| | |
|---|---|
| Keywords: | MENTAL HEALTH, Other metabolic, e.g. iron, porphyria < DIABETES & ENDOCRINOLOGY, NUTRITION & DIETETICS |



SCHOLARONE™
Manuscripts

# Healthy Early Life Moments in Singapore (HELMS): study protocol for a single-arm pilot implementation model

Jerry Kok Yen Chan[1,2, 3], Chee Wai Ku[1,2], See Ling Loy[1,2], Keith M. Godfrey[4,5], Yiping Fan[1], Mei Chien Chua[2,6], Fabian Yap[3,7,8]

[1]Department of Reproductive Medicine, KK Women's and Children's Hospital, Singapore, Singapore 229899

[2]Duke-NUS Medical School, Singapore, Singapore 169857

[3]Yong Loo Lin School of Medicine, National University of Singapore, Singapore, Singapore 119228

[4]Medical Research Council Lifecourse Epidemiology Centre, University of Southampton, Southampton SO16 6YD, United Kingdom

[5]National Institute for Health Research Southampton Biomedical Research Centre, University of Southampton and University Hospital Southampton National Health Service Foundation Trust, Southampton SO16 6YD, United Kingdom

[6]Department of Neonatology, KK Women's and Children's Hospital, Singapore, Singapore 229899

[7]Department of Paediatrics, KK Women's and Children's Hospital, Singapore, Singapore 229899

[8]Lee Kong Chian School of Medicine, Nanyang Technological University, Singapore, Singapore 636921

Corresponding Authors: Jerry Kok Yen Chan and Fabian Yap

For peer review only

Department of Reproductive Medicine, KK Women's and Children's Hospital, Singapore, Singapore 229899

jerry.chan.k.y@singhealth.com.sg

Department of Paediatrics, KK Women's and Children's Hospital, Singapore, Singapore 229899

fabian.yap.k.p@singhealth.com.sg

For peer review only

Word count: 4567

Keywords: Obesity, metabolic health, mental health, lifestyle intervention, preconception

## ABSTRACT

### Introduction

Changes in social and lifestyle factors have led to increasing rates of metabolic and mental health problems. We hypothesize that a transformation of the current maternal and child health system is required to deliver interventions that effectively promote a good start to life in populations at risk of metabolic and mental health problems. We describe a pilot implementation model "Healthy Early Life Moments in Singapore" (HELMS), which aims to examine whether an integrated lifestyle intervention initiated at preconception and continuing throughout pregnancy and postpartum periods can improve the metabolic and mental health of overweight and obese women, and improve early child growth.

### Methods and Analysis

This single-arm pilot implementation trial aims to recruit 500 women aged 21-40 years with a body mass index of 25-40 kg/m$^2$ who plan to get pregnant, with interventions delivered before conception, until 18 months post-delivery. Primary outcomes comprise pregnancy rate, maternal metabolic and mental health status. Secondary outcomes include maternal reproductive health, pregnancy outcomes, and offspring growth. The intervention will be delivered on a digital platform, to provide anticipatory guidance, raise awareness, and guide goal-setting on lifestyle behaviours that include diet, physical activity, mental wellness, and sleep hygiene from preconception to postpartum. Women who conceive within one year of recruitment will be followed through pregnancy and studied with their infants at six time points during the first 18 months of life. Questionnaires, anthropometric measurements and multiple biosamples will be collected at each visit. This study will pave the way for novel models of care that adopt a holistic intervention throughout the early life-course, with greater integration between primary and tertiary healthcare systems and ongoing community efforts to engage and support women in this journey.


For peer review only



For peer review only - http://bmjopen.bmj.com/site/about/guidelines.xhtml

## Ethics and Dissemination

The study has been approved by the Centralised Institutional Review Board of SingHealth (2021/2247) and ClinicalTrials.gov (NCT05207059).

## ARTICLE SUMMARY

### Strengths and limitations of this study

- HELMS aims to examine the impact of integrated interventions, initiated preconceptionally and continued throughout the pregnancy and postpartum phases, on metabolic and mental health of women who are overweight or obese.
- Extensive biosampling and detailed phenotyping of mother, father, and child will allow longitudinal assessment of changes in health behaviours including diet, physical activity and sleep, maternal metabolic profile, and depression risk.
- This will provide an important platform for biomarker discovery, validate integrated HELMS interventions, and pave the way for new guidelines for lifestyle programs from preconception to postpartum stages.
- The main limitation of this study design include the inability to distinguish between the effect of intervention, a placebo effect, and the effect of natural history.
- HELMS is a single-centre study of the Asian population, involving only English-speaking participants, so external validity may be limited to this population, and caution should be exercised in generalizing results across different settings and populations.

For peer review only

## INTRODUCTION

Non-communicable diseases (NCDs) represent the predominant cause of morbidity and mortality worldwide.[1] Rapid urbanization, unhealthy diet and sedentary lifestyle have led to an epidemic of metabolic diseases, which are the main drivers of NCDs.[1] Coupled with this metabolic epidemic is a rising rate of mental disorders, especially depression, as the leading cause of disability worldwide that commonly affects women.[2] Both metabolic and mental disorders are interrelated, with their co-occurrence frequently observed in individuals living with obesity.[3 4] The short- and long-term health risks of maternal obesity and depression in mothers and their children are well documented.[5–7] Women living with obesity and mental health conditions are at increased risk of infertility, adverse obstetric outcomes, and postpartum cardiometabolic complications; and their children are also susceptible to obesity, metabolic disease, and psychosocial disturbances in childhood and adolescence.[5–7] In Singapore, a quarter of women are overweight or obese [body mass index (BMI) ≥25 kg/m$^2$] before pregnancy,[8] while one in ten women experience depression before, during and after pregnancy.[9 10]

To mitigate the impact of maternal obesity and depression, various intervention strategies targeting at antenatal and postpartum periods have been studied. These include setting SMART (Specific, Measurable, Achievable, Relevant, Time-Bound) goals, providing healthy lifestyle counselling or group-based education, perinatal mental health interventions, and providing health service support.[11–13] However, these antenatal- and immediate postpartum-phase focused interventions have had modest success,[14 15] only some showing improvements in eating behaviour, physical activity or weight status,[11 16–18] and most failing to prevent adverse mother-offspring outcomes such as gestational diabetes mellitus (GDM) and macrosomia. This could be attributed to the relatively short duration of the intervention, the



For peer review only - http://bmjopen.bmj.com/site/about/guidelines.xhtml

lack of continued care from preconception, and intervention only beginning in the antenatal and postpartum periods, which may be too late to produce any meaningful impact.

There is increasing focus on interventions during the preconception period, especially for women living with obesity, who are susceptible to both metabolic and mental disorders.[3 19] Such interventions not only improve the health of the mother, but also provide potential health benefits to the next generation through an improved environment for embryo and foetal development.[20 21] The preconception period represents a unique opportunity where women have the greatest motivation to make a positive change.[22] So far, preconception lifestyle interventions have demonstrated positive maternal behaviour changes, such as increased intake of multivitamins, vegetables and other dietary changes, increased physical activity, as well as smoking cessation and reduced alcohol consumption, resulting in reduction in BMI and gestational weight gain (GWG), higher clinical pregnancy rate and lower preterm delivery.[23–25] This has led to improved knowledge, self-efficacy, and control.[26] Furthermore, women who achieved at least 5% weight loss during such interventions had better cardiometabolic health based on glycaemic and lipid measures six years later.[25]

Taken together, this suggests that adopting a life-course perspective in the healthcare model with a continuum of care provided before, during, and after pregnancy has the potential to address the trajectory of increased metabolic and mental disorder risk throughout these life stages. Acting upstream before conception to optimize health is crucial to improving reproductive potential while reducing the social and health implications resulting from unplanned pregnancies. Modifying current antenatal care services by empowering women in preparation for pregnancy and childbirth is critical for the wellbeing of both mother and baby. Optimizing postpartum recovery and readiness, and nurturing infants through optimal feeding



For peer review only - http://bmjopen.bmj.com/site/about/guidelines.xhtml

practices and growth monitoring are important for promoting virtuous life cycles of health in an individual and breaking vicious life cycles of NCDs for the next generation.[27]

## GOALS AND AIMS

We envision an integrated life-course approach with a continuum of care that encompasses preconception preparation, pregnancy optimization, along with postpartum synchronization of maternal-child health services in the first 18 months of life. Connecting the preconception, pregnancy and postpartum journeys will achieve synergism in producing greater behavioural change and beneficial outcomes not only for women, but also for the child and family.[19] Therefore, the goals of Healthy Early Life Moments in Singapore (HELMS) are (**i**) to develop a life-course model of care (MOC) focusing on preventive healthcare starting from preconception, a time where the cost effectiveness of intervention is likely to be maximum,[28] and (**ii**) to implement the MOC that represents the clinical translation of decades of scientific evidence related to early developmental programming from preconception to postpartum, based on the Developmental Origins of Health and Disease (DOHaD) paradigm.[20]

In this study, the overarching aim is to examine whether an integrated intervention beginning preconceptionally and continuing throughout the pregnancy and postpartum phases can improve the metabolic and mental health of women living with obesity, as well as improve offspring growth and development. At each life-course phase, the study has specific goals to achieve, including (1) improving fertility during preconception; (2) optimizing obstetric outcomes during pregnancy; (3) improving postpartum physical and mental wellbeing, and promoting healthy feeding habits and optimal growth during infancy. We will examine changes of maternal metabolic profile, risk of depression, health behaviours including diet,



For peer review only - http://bmjopen.bmj.com/site/about/guidelines.xhtml

physical activity, and sleep over time. An overview of the study framework is shown in **Figure 1**.

## METHODS

### Trial Design and Setting

This study represents a model of pilot implementation to demonstrate the value of adopting a life-course approach and integrated care to maternal-child health. It is conceptualized as a single-arm pilot trial, with a targeted intervention implemented in a group of women living with obesity who are trying to conceive within one year after recruitment. Longitudinal intervention will be performed from preconception, throughout pregnancy until 18 months postpartum in the KK Women's and Children's Hospital (KKH), Singapore. The protocol is written in accordance with the SPIRIT guidelines. Written informed consent will be obtained from all participants at the recruitment.

The flow of the trial is shown in **Figure 2**. Participants will be reviewed at multiple time points from preconception through pregnancy, and both participants and their infants will be followed for the first 18 months after delivery, with extensive biosampling and detailed phenotyping performed longitudinally. Following informed consent at the first preconception clinic visit, participants will complete a set of questionnaires that assess baseline demographics, diet practices, physical activity, sleep, emotion, and sexual health. Anthropometric measurements will be taken, together with biosample collection, including blood for a standard 75g oral glucose tolerance test (OGTT) and stool. Participants will receive a doctor's consultation, along with prescription of a standard preconception supplement, digitized preconception care and healthy lifestyle guidance. The second preconception clinic visit will take place three months later, with questionnaires and


For peer review only - http://bmjopen.bmj.com/site/about/guidelines.xhtml

measurements being performed, and compliance to the intervention being reviewed. Phone contact will be made six and nine months after recruitment to help participants maintain focus on their preconception health and lifestyle goals, and to remind them to perform a urine pregnancy test if periods are missed. If they do not become pregnant, an exit visit will be conducted at 12 months with questionnaires, measurements, and biosampling, followed by appropriate evaluations and subfertility referrals thereafter. For participants who become pregnant, they will be seen between 6-10 weeks of gestation at the first pregnancy visit. Questionnaires, measurements, and biosampling will again be performed. The second HELMS pregnancy visit will be at 24-28 weeks, and the final HELMS visit in pregnancy will be at 32-36 weeks. Continuous antenatal care and healthy lifestyle guidance during pregnancy will be provided throughout the trimesters. At delivery, maternal and infant biosampling, including placenta collection, will be performed. In the postpartum dyad phase, there will be 6 visits over an 18-month period, according to the infant vaccination schedule, at 1-2 weeks, 6-8 weeks, 4, 6, 12, and 18 months. An ongoing intervention tailored to this postpartum dyad phase will be delivered, together with questionnaires, measurements, and biosampling for mother and child at each visit. Throughout the study, participants will be asked to log their weight and supplement intake weekly in a digital calendar. For the partner, anthropometric measurements will be collected once during preconception, pregnancy, and postpartum periods while blood will be collected once throughout the entire study period. Meanwhile, partners will be passively engaged in the intervention by receiving the same set of digitized intervention materials. A feedback survey on the HELMS program will be performed at the end of each preconception, pregnancy, and postpartum phases.

For peer review only

### Participants and recruitment

Potential participants from the public or KKH (patients/ staff) or referred from other healthcare institutes who fulfil the inclusion criteria will be invited to participate in the HELMS study. KKH houses the largest Obstetrics and Gynaecology department in Singapore, managing approximately 12,000 deliveries a year with patients made up of a wide sociodemographic spectrum. Invitation letters, emails, messages, webpages, brochures, and posters will be used for advertising. Interested women can contact the study team by email or phone.

Inclusion criteria include (1) woman who plans to conceive in the next 12 months; (2) BMI 25-40 kg/m$^2$; (3) age 21-40 years; (4) Chinese, Malay, Indian ethnicity, or any combination of these three ethnic groups; (5) intending to reside in Singapore for the next four years; (5) can access the Internet through any digital platform; and (6) can provide written informed consent. Exclusion criteria include (1) currently pregnant; (2) difficulty in understanding the English language; (3) known Type 1 or Type 2 diabetes; (4) on anticonvulsant medication, oral steroid, contraception, or fertility medication in the past one month; (5) on HIV or Hepatitis B or C medication in the past one month. If a woman has a pregnancy loss and wishes to re-join the study, she will be re-characterized as at the preconception baseline visit at least one month after a negative urinary pregnancy test and will receive intervention as before.

Women who suffer from a miscarriage or termination event, experience multiple pregnancies, with foetal congenital anomalies, cannot comply with the study protocol or wish to discontinue their participation will be withdrawn from the study.


For peer review only - http://bmjopen.bmj.com/site/about/guidelines.xhtml

**Intervention overview**

All participants will receive an intervention via a digital platform, designed to address and improve women's knowledge, attitude, and practice in terms of preconception-pregnancy-postpartum care and health behaviours. There will be four modules, namely the HELMS Journey, the HELMS Model, the HELMS Lifestyle, and the HELMS Community. The HELMS Journey will provide anticipatory guidance on examinations and measurements performed during clinic visits. The HELMS Model will deliver the 4S (Screening, Size, Supplementation, Special considerations) care plans. The HELMS Lifestyle will provide support in healthy eating, physical activity, mental wellness, and sleep hygiene. Finally, the HELMS Community will provide social support and improve participation in the program. Each of these modules contains phase-specific information developed by obstetricians, neonatologists, paediatricians, dietitians, physiotherapists, psychiatrists, and psychologists.

**Intervention Details**

*1. HELMS Journey*

Details of each preconception, pregnancy, and postpartum visit of HELMS will be available here to provide anticipatory guidance on these visits. This will ensure that participants are aware of the study measurements, as well as the required routine examinations.

*2. HELMS Model*

The HELMS life-course interventions, namely 4S, are developed to provide care throughout the journey from preconception to postnatal. 4S is represented by '**S**creening', '**S**ize', '**S**upplementation' and '**S**pecial considerations'.

- **Screening** involves health and risk assessments through physical and biomarker (e.g. Anti-Mullerian hormone, glucose, insulin and lipid profile) measurements, as well as



For peer review only - http://bmjopen.bmj.com/site/about/guidelines.xhtml

emotion and sleep evaluations for mothers, and developmental assessment for children.

- Body **Size** management encompasses education on weight status awareness and mother-child health implications, both mother-child weight tracking, healthy eating, and physical activity guidance.
- **Supplementation** includes multi-micronutrient, vitamin D, calcium, and / or DHA supplements, which are phase specific for mother, and vitamin D supplement drops for the child.
- **Special considerations** include the management of preconception sexual health and function, the management of pregnancy symptoms and postpartum recovery, and the monitoring of infant growth and feeding management.

*3. HELMS Lifestyle*

Metabolic health support (Diet and Physical activity modules)

The **diet module** is developed based on the 6P tool, which is designed as a platform to address metabolic health based on the principles of energy input and expenditure, as well as motivation as the basis for dietary change.[29] It is a simple, self-administered instrument based on the concept of a mental model, which can lead to self-awareness, self-evaluation, and self-education, over time resulting in a positive change in eating habits and health. The 6P comprises 6 components as presented in **Table 1**. Multiple modalities are incorporated that include feedback on 6P behaviour, 6P self-monitoring, 6P goal setting, and 6P nudges along with the use of the 6P tool. By including these 6 main discrete dietary and activity components under a single 'package', it will provide an overview of the mental model of nutrition based on the principle of energy input and expenditure, promoting self-awareness of unhealthy lifestyle behaviours and nudging individuals into actual implementation via

For peer review only

concrete, personalized feedback and increasing intrinsic motivation. This is based on the theoretical framework of the Theory of Planned Behaviour (TPB) which has been widely used to predict behavioural intention and health-related behaviors.[30]

**Table 1** Components of the 6P tool

| **6P** | **Healthy mental model of nutrition** | **Description** |
|---|---|---|
| P1 | PORTION | Amount of food intake |
| P2 | PROPORTION | Caloric density of food intake |
| P3 | PLEASURE | Frequency of snacks and sugary beverages and irregular intake |
| P4 | PERIOD | Time of day of food intake |
| P5 | PHYSICALITY | Physical activity and sedentary behaviour |
| P6 | PSYCHOLOGY | Readiness for change |

The **physical activity module** seeks to engage women with the importance, type, and intensity of exercise during each phase. It consists of a series of appropriate, tailored exercise videos developed by physiotherapists and obstetricians with the support of scientific evidence. Wearable activity trackers will be used to provide real-time feedback on physical performance and help participants monitor their energy expenditure. Sustainability will be supported with the use of nudges (both text messages and images) delivered weekly to influence behaviour and decision-making.

Mental health support (Mental health and Sleep modules)

For peer review only



For peer review only - http://bmjopen.bmj.com/site/about/guidelines.xhtml

The **mental health module** serves as the main way to support, screen, and guide women's mental health longitudinally. Upstream screening of depressive symptoms at the preconception stage allows women at high risk to be triaged earlier for receiving targeted mental health support from KKH's mental health team comprising a clinical coordinator, case manager and psychiatrist. Women at medium risk will be engaged more intensively, while those at low risk will continue to participate, enabling and preparing for different stages through a package of clinical care and lifestyle support, especially in the late stages of pregnancy and early stages of postpartum. The **sleep module** aims to support mental wellness by promoting healthy sleep practices throughout the life-course. A wearable sleep tracker will be used to provide real-time feedback on sleep performance and help participants to monitor their sleep patterns.

*4. HELMS Community*

An online community platform is being established as part of supporting mental health among HELMS participants. This allows women to share their experience, identify common problems among peers and generate solutions that work for them in the online chat group.

Our intervention approach will be based on the **SIGN strategy** (Support, Inform, Guide and Nudge) to deliver the 4S and healthy lifestyle support. Close monitoring of women's health allows timely **support** provided by the healthcare staff. Setting up mobile health education helps to **inform** women about the importance and warning points in different stages. Educational materials in various formats, including diagrams and videos, provide **guidance** on phase-specific care and healthy lifestyles that focus on nutrition, physical activity, mental health, and sleep hygiene. Health **nudges** in the form of key messages related to preconception, pregnancy, and postpartum dyad care, as well as healthy lifestyles, facilitate



For peer review only - http://bmjopen.bmj.com/site/about/guidelines.xhtml

care support and behavioural changes. The use of nudges represents a preferred architecture strategy that is widely used in public policy making, to alter people's behaviour and influence decision making.[31]

In summary, we will apply an interactive and personalized approach to conducting the intervention, supported by real-time feedback. This involves goal setting, individual education, self-awareness, self-monitoring, motivation, and outcome review process. These techniques are commonly used in behavioural interventions and can improve health outcomes.[32 33]

**Intervention adherence**

Adherence to the intervention will be established throughout the study with regular logbook charts of supplement intake, sexual activity, and weight, which will be reviewed at each study visit or by telephone reminders. The use of a digital tracker for activity and sleep allows off-site monitoring of wearing adherence, and research staff will take action to check for reasons for non-adherence.

**Outcomes and assessments**

The primary outcome is the pregnancy rate in overweight and obese women trying to conceive within one year of baseline assessment. It is defined by a positive urinary pregnancy test, followed by ultrasound confirmation of an intrauterine gestational sac after six weeks of amenorrhea. If an ultrasound scan is not available or inconclusive, the diagnosis of pregnancy will be made clinically. A successful conception is one of the most important clinical outcomes, and it represents the culmination of improved metabolic and mental health, leading to optimized gametogenesis.



For peer review only - http://bmjopen.bmj.com/site/about/guidelines.xhtml

Co-primary outcomes include maternal metabolic health and mental health status. Maternal metabolic health is assessed by metabolic syndrome criteria. Mental health is evaluated using the Edinburgh Postnatal Depression Scale (EPDS).[34]

Other key outcomes include:

**Women/mothers**

- Reproductive health as assessed by fecundability, pregnancy loss and live birth rates, and sexual function based on Female Sexual Function Index-19.[35]
- Pregnancy outcomes include pain, obstetric, and delivery complications.
- Health behaviours assessed by dietary practice (6P tool,[29] 4-day food diary, Food Frequency Questionnaire,[36] Three Factor Eating Questionnaire,[37] alcohol and supplement intake), smoking exposure, physical activity, and sedentary behaviours (International Physical Activity Questionnaire,[38 39] Sedentary Behaviour Questionnaire,[40] accelerometer), and sleep (Pittsburgh Sleep Quality Index,[41] digital tracker).
- Metabolic profile, including weight status, body fat distribution, blood pressure, lipid, and glycaemic measures
- Gut microbiome profile assessed by stool sample.
- Nutrient status based on diet, blood, and breast milk composition.
- Ocular health as assessed by retinal vessel calibre characteristics

**Offspring**

- Anthropometry and growth assessed by antenatal serial ultrasound scans, infant weight, length, and head circumference.



For peer review only - http://bmjopen.bmj.com/site/about/guidelines.xhtml

- Neonatal complications such as hypoglycaemia and admission to neonatal care facilities; infant health and well-being, and skin biopsy for a subset of infants with eczema.
- Infant feeding assessed by breastfeeding behaviours, time of weaning, nutrition milestone, dietary intake and eating behaviours.

**Partners**

- Physical and metabolic health assessed by weight, BMI, and body fat distribution
- Health behaviours assessed by smoking exposure, alcohol intake, meal pattern, stress level, physical activity, and sedentary behaviours

Program effectiveness will be evaluated based on women's quality of life, healthcare utilization and participant feedback surveys. Blood, cord blood, stool, urine, saliva, placenta, and breast milk samples will be stored for analyses on biochemical, micronutrient, metabolomic, genomic, epigenetic, immunological, and molecular profiles.

**Patients and public involvement**

We have conducted in-depth face-to-face interviews with preconception, pregnant and postpartum women who were overweight and obese to understand their needs throughout the preconception-pregnancy-postpartum journey, enablers and barriers to a healthy lifestyle, intervention preferences, and delivery methods. This work enabled development of the intervention package and dissemination plans.[42]



For peer review only - http://bmjopen.bmj.com/site/about/guidelines.xhtml

**Planned analyses**

Continuous variables will be presented as means and standard deviations, or medians and 25th – 75th centiles, as appropriate. Categorical variables will be presented as numbers and percentages. The pregnancy rate will be determined by the number of women who became pregnant (defined by stated previously) divided by the total number of women who completed the 1-year follow-up during the preconception phase. The time to pregnancy (TTP) will be estimated by the number of menstrual cycles required to achieve a pregnancy over one year of follow-up. We will use the discrete-time proportional hazards model, which analyses TTP as a discrete scale based on the number of menstrual cycles, to estimate the fecundability ratio (FR) and the 95% confidence interval (CI),[43 44] accounting for left truncation and right censoring. For the other two co-primary outcomes, linear mixed effects model will be used to examine changes in means between the baseline and follow-up metabolic markers and EPDS scores, with adjustment for baseline potential confounders and duration of the intervention received. In addition to the pre-post comparison of outcomes stated above, differences with pregnancy and birth outcomes will also be compared with a similar observational cohort in Singapore called Singapore PREconception Study of long-Term maternal and child Outcomes (S-PRESTO),[45] using multiple linear or logistic regression models, adjusting for potential confounders. These confounders, such as age, ethnicity, and education, will be determined from literature review, directed acyclic graph and/or observed statistically significant associations with exposures and outcomes.

We will impute missing data using multiple imputation analyses by chained equations.[46] The number of imputations will be determined based on percentage of missing values,[47] and results of total imputations will be pooled using Rubin's rule.[48] To determine whether imputation of the missing data may have affected the results, we will perform sensitivity analyses on participants with a complete set of data.



For peer review only - http://bmjopen.bmj.com/site/about/guidelines.xhtml

**Sample size calculation**

Based on the S-PRESTO study,[45] which is an observational cohort recruited from preconception and followed up through postpartum periods, preconception overweight or obese women (BMI ≥25 kg/m$^2$) had a 38% pregnancy rate, while those with normal weight (BMI 18.5 - 24.9) had 47% pregnancy rate. Among overweight and obese women, those with healthy metabolic profile characterised by absence of the metabolic syndrome or insulin resistance had a 52% pregnancy rate.[49] We anticipate that HELMS intervention will improve overweight or obese women's pregnancy rate from 38% to 50% within 12 months of trying. Using a two-sided α level of 5% and a power of at least 90%, 400 participants are needed in the preconception phase, leading to 200 pregnancies. Considering a 20% dropout rate, 500 participants are required for recruitment. We estimate that 15% of women will experience pregnancy loss, leaving 170 dyad pairs to be followed.

For peer review only



For peer review only - http://bmjopen.bmj.com/site/about/guidelines.xhtml

**DISCUSSION**

The existing MOC in maternal-child health in Singapore and in many developed countries emphasizes disease treatment with less emphasis on health promotion. Most women are not prepared for pregnancy, and many have poor metabolic and mental health. This lack of preparedness carries over in the postpartum phase after the child is born. A woman's own needs are often neglected in the immediate postpartum phase, when the attention is primarily focused on caring for the newborn. Coupled with unexpected physical and emotional challenges in this new phase of life, it culminates in a cycle of poor metabolic and mental health. Women living with overweight and obesity represent a particularly high-risk group for poor metabolic and mental health.[50 51] The current obesity management strategy has three fundamental gaps. Firstly, the timing of intervention is too far downstream in adulthood. Secondly, there is weak foundational knowledge of nutrition and physical activity, with poor awareness and insight about metabolic health. Thirdly, there is no structured and coordinated care plan for these women while planning to conceive, which would be an ideal window for intervention during the crucial period of gametogenesis.[52] To address these challenges, there is a need to invert the pyramid of care and focus our effects upstream, starting from preconception.

HELMS aims to address the twin challenges of metabolic and mental health disorders through a series of interventions, with the goal of providing the best and most equal start to life for children. Through its life-course approach, HELMS recognises the additive effects of influences on a person's life that will impact the future health trajectory. It aims to impart new mental models of nutrition, physicality, and mental wellness, by guiding, supporting, and empowering women through an integrated journey from preconception to postpartum, to address metabolic and mental health challenges. We hope this unique holistic approach with



For peer review only - http://bmjopen.bmj.com/site/about/guidelines.xhtml

a continuum of care across the early life-course, starting from preconception, pregnancy until the postpartum period will build a strong foundation for healthy women and secure a healthy start for future generations.

Drawing on the experience and evidence derived from two local prospective observational mother-offspring cohorts conducted at KKH and National University Hospital (NUH) since 2008 – Growing Up in Singapore Toward Healthy Outcomes (GUSTO) and the S-PRESTO),[45 53] HELMS seek to examine the impact of integrated interventions, initiated preconceptionally, on metabolic and mental health of women living with overweight or obesity. Extensive biosampling and detailed phenotyping of mother, father, and child will allow longitudinal assessment of changes in health behaviours including diet, physical activity and sleep, maternal metabolic profile, and depression risk. The biosamples will provide an important platform for biomarker discovery, validate integrated HELMS interventions, and pave the way for new guidelines for lifestyle programs from the preconception to postpartum stages.

Despite the strong evidence base for adverse maternal and child outcomes in overweight and obese women, as well as the benefit of lifestyle interventions in the preconception and pregnancy phases, integrated HELMS interventions are novel and have not established unequivocal evidence of benefit. Thus, HELMS is conceptualized as a pilot single arm intervention trial in women living with overweight or obesity to obtain preliminary evidence of efficacy of intervention. A subsequent larger pragmatic trial is envisaged to validate the efficacy of the intervention. The main limitations of this study design include the inability to distinguish between the effect of intervention, a placebo effect, and the effect of natural history, including regression to the mean. Given the similarity in study design to the



For peer review only - http://bmjopen.bmj.com/site/about/guidelines.xhtml

prospective observational cohort S-PRESTO, with similar time points of follow-up and biosampling, there is the potential for comparison with this historical cohort. HELMS is a single-centre study of the Asian population, involving only English-speaking participants, thus, the external validity may be limited to this population, and caution will need to be exercised in generalizing results across different settings and populations. Finally, the interventions are tailored to the woman / mother, with only passive intervention for her partner. The environment of the woman plays an important role in her compliance to lifestyle interventions,[54] which may undermine the impact of our program. On the other hand, if the success of the HELMS interventions extends to that of her immediate family, we would also have limited insights with minimal measurements on the partner, to reduce the burden of involvement in the study.

## ETHICS APPROVAL

The study has been approved by the Centralised Institutional Review Board of SingHealth (2021/2247) and registered on ClinicalTrials.gov (NCT05207059).

### Quality control

Procedures and actions will be implemented throughout the study to ensure that information provided to all participants is standardized, data collected is as complete as possible, and of high quality. Research staff responsible for recruiting and follow-up participants will receive training from study leads on recruitment, consent taking, questionnaire and data management, intervention delivery and compliance monitoring. An operation database will be developed to monitor participant progression throughout the study, schedule study visits and monitor visit/measure completeness. A quarterly meeting will be held among study leads and research


For peer review only - http://bmjopen.bmj.com/site/about/guidelines.xhtml

staff to review recruitment process, intervention delivery, data collection and participants' feedback. An annual audit on the study will be performed by an independent party.

**Data monitoring**

All data will be pseudonymised. Participants' identifiers will be kept separately in a password-protected file and only be assessed by specific research staff. Electronic data will be managed using a secure, encrypted online data capturing system approved by the institution. A data monitoring team will perform data checking on completeness, errors and outliers. To facilitate data monitoring procedure, algorithms are programmed to auto detect implausible value while entering data, and to prompt message for incomplete entry. Ad hoc discussions will be organized with study leads to clarify actions required to resolve data issues.

**Trial Status**

HELMS recruitment is planned to begin in March 2022 and be completed in September 2023. The current protocol is version 1, dated 16 Dec 2021.

**AUTHORS CONTRIBUTIONS**

Conceptualization, J.Y.K.C, F.Y, C.W.K and S.L.L; methodology, J.Y.K.C, F.Y, K.M.G, C.W.K and S.L.L; writing—original draft preparation, C.W.K, S.L.L and Y.F; writing—review and editing, all authors.; funding acquisition, M.C.C, F.Y and J.K.Y.C. All authors have read and agreed to the published version of the manuscript.

For peer review only

**FUNDING STATEMENT**

This research was supported by the KKH Health Services Model of Care Transformation Fund (MoCTF) Grant (MoCTF/01/2020, MoCTF/02/2020 and MoCTF/03/2020) and the Lien Foundation Optimising Maternal and Child Health Programme Fund (Grant number not applicable). The funding body did not influence either the data collection and analysis or the writing and the decision to submit the manuscript. C.W.K. and J.K.Y.C. are supported by the National Medical Research Council, Ministry of Health, Singapore (NMRC/MOH-000596-00 and NMRC/CSA-SI-008-2016, respectively). K.M.G. is supported by the National Institute for Health Research (NIHR Senior Investigator (NF-SI-0515-10042), NIHR Southampton 1000DaysPlus Global Nutrition Research Group (17/63/154) and NIHR Southampton Biomedical Research Center (IS-BRC-1215-20004), British Heart Foundation (RG/15/17/3174) and the European Union (Erasmus+ Programme ImpENSA 598488-EPP-1-2018-1-DE-EPPKA2-CBHE-JP).

**COMPETING INTERESTS STATEMENT**

K.M.G. and F.Y. received reimbursement for speaking at conferences sponsored by companies that sell nutritional products. K.M.G. is part of an academic consortium that received research funding from Abbott Nutrition, Nestle and Danone. All other authors declare no competing interests.

**ACKNOWLEDGEMENTS**

The authors would like to thank KK Women's and Children's Hospital for the institutional support received during this study. We would also like our multi-disciplinary team of collaborators including, but not limited to, Dr Helen Chen, Dr Han Wee Meng, Dr Mary Chong, Dr Jacinth Tan, Dr Patrick Tan, Dr Saumya Jamuar, Dr Salvatore Albani, Dr Florent

For peer review only

Ginhoux Dr Joanne Yoong, Dr Ng Wei Yen, Dr Jeremy Lim, Dr Michael Chee, Dr Dan Yock Young, Dr Falk Mueller-Riemen-Schneider, and Dr Lynette Shek. The authors also thank the contributions of Ms Ng Xiang Wen, Ms Lai Lan Tian, Ms Rachael Loo Si Xuan, and Ms Faith Liew Hui Hua for their administrative support.

## FIGURES

**Figure 1**. The Healthy Early Life Moments in Singapore (HELMS) framework, comprising preconception, pregnancy, and postpartum dyad care. Through maternal behavioural intervention, the overarching aim is to improve metabolic and mental health in women living with obesity, and to improve early child growth. Specifically, HELMS aims to improve women's fertility, obstetric outcomes, physical and mental recovery from birth, feeding habits and growth of the child.

**Figure 2.** HELMS study flow diagram. Preconception visits are coloured green, pregnancy visits pink, and postpartum visits blue. HELMS, Healthy Early Life Moments in Singapore.

For peer review only

## REFERENCES

1. GBD 2015 Risk Factors Collaborators. Global, regional, and national comparative risk assessment of 79 behavioural, environmental and occupational, and metabolic risks or clusters of risks, 1990–2015: a systematic analysis for the Global Burden of Disease Study 2015. *Lancet* 2016;388:1659–1724.
2. World Health Organization. *Depression*. 2021. https://www.who.int/news-room/fact-sheets/detail/depression. [Accessed 29 Sep 2021].
3. Kim SR, Kim HN, Song SW. Associations Between Mental Health, Quality of Life, and Obesity/Metabolic Risk Phenotypes. *Metab Syndr Relat Disord* 2020;18:347–52.
4. Limon VM, Lee M, Gonzalez B, *et al*. The impact of metabolic syndrome on mental health-related quality of life and depressive symptoms. *Qual Life Res* 2020;29:2063–72.
5. Poston L, Caleyachetty R, Cnattingius S, *et al*. Preconceptional and maternal obesity: epidemiology and health consequences. *Lancet Diabetes Endocrinol* 2016;4:1025–36.
6. Becker M, Weinberger T, Chandy A, *et al*. Depression During Pregnancy and Postpartum. *Curr Psychiatry Rep* 2016;18:32.
7. Dalrymple KV, El-Heis S, Godfrey KM. Maternal weight and gestational diabetes impacts on child health. *Current Opinion in Clinical Nutrition and Metabolic Care* – in press.
8. Loy SL, Cheung YB, Soh SE, *et al*. Female adiposity and time-to-pregnancy: a multiethnic prospective cohort. *Hum Reprod* 2018;33:2141–49.
9. Kee MZL, Ponmudi S, Phua DY, *et al*. Preconception origins of perinatal maternal mental health. *Arch Womens Ment Health* 2021;24:605–18.
10. Law EC, Aishworiya R, Cai S, *et al*. Income disparity in school readiness and the mediating role of perinatal maternal mental health: a longitudinal birth cohort study. *Epidemiol Psychiatr Sci* 2021;30:e6.

For peer review only

11. Poston L, Bell R, Croker H, *et al*. Effect of a behavioural intervention in obese pregnant women (the UPBEAT study): a multicentre, randomised controlled trial. *Lancet Diabetes Endocrinol* 2015;3:767–77.

12. Lavender TJ, Ebert L, Jones D. An evaluation of perinatal mental health interventions: An integrative literature review. *Women Birth* 2016;29:399–406.

13. Hurt L, Paranjothy S, Lucas PJ, *et al*. Interventions that enhance health services for parents and infants to improve child development and social and emotional well-being in high-income countries: a systematic review. *BMJ Open* 2018;8:e014899.

14. Thangaratinam S, Rogozinska E, Jolly K, *et al*. Effects of interventions in pregnancy on maternal weight and obstetric outcomes: meta-analysis of randomised evidence. *BMJ* 2012;344:1–15.

15. Rogozińska E, Chamillard M, Hitman GA, *et al*. Nutritional manipulation for the primary prevention of gestational diabetes mellitus: a meta-analysis of randomised studies. *PloS One* 2015;10:1–21.

16. Dodd JM, Cramp C, Sui Z, *et al*. The effects of antenatal dietary and lifestyle advice for women who are overweight or obese on maternal diet and physical activity: the LIMIT randomised trial. *BMC Med* 2014;12:161.

17. Simmons D, Devlieger R, van Assche A, *et al*. Effect of Physical Activity and/or Healthy Eating on GDM Risk: The DALI Lifestyle Study. *J Clin Endocrinol Metab* 2017;102:903–13.

18. Okesene-Gafa KAM, Li M, McKinlay CJD, *et al*. Effect of antenatal dietary interventions in maternal obesity on pregnancy weight-gain and birthweight: Healthy Mums and Babies (HUMBA) randomized trial. *Am J Obstet Gynecol* 2019;221:152.e1–152.e13.



For peer review only - http://bmjopen.bmj.com/site/about/guidelines.xhtml

19. Hanson M, Barker M, Dodd JM, *et al*. Interventions to prevent maternal obesity before conception, during pregnancy, and post partum. *Lancet Diabetes Endocrinol* 2017;5:65–76.

20. Hanson MA, Gluckman PD. Developmental origins of health and disease: moving from biological concepts to interventions and policy. *Int J Gynaecol Obstet* 2011;115:S3–5.

21. Stephenson J, Schoenaker D, Hinton W, Poston L, Barker M, Alwan N, Godfrey K, Hanson M, de Lusignan S. A 'wake-up call' for preconception health: a clinical review. *Br J General Practice* 2021; 71:233-236.

22. Barker M, Dombrowski SU, Colbourn T, *et al*. Intervention strategies to improve nutrition and health behaviours before conception. *Lancet* 2018;391:1853–64.

23. Lan L, Harrison CL, Misso M, *et al*. Systematic review and meta-analysis of the impact of preconception lifestyle interventions on fertility, obstetric, fetal, anthropometric and metabolic outcomes in men and women. *Hum Reprod* 2017;32:1925–40.

24. Jacob CM, Newell M-L, Hanson M. Narrative review of reviews of preconception interventions to prevent an increased risk of obesity and non-communicable diseases in children. *Obes Rev* 2019;20:5–17.

25. Wekker V, Huvinen E, van Dammen L, *et al*. Long-term effects of a preconception lifestyle intervention on cardiometabolic health of overweight and obese women. *Eur J Public Health* 2019;29:308–14.

26. Hussein N, Kai J, Qureshi N. The effects of preconception interventions on improving reproductive health and pregnancy outcomes in primary care: A systematic review. *Eur J Gen Pract* 2016;22:42–52.

27. Bütikofer A, Løken KV, Salvanes KG. Infant health care and long-term outcomes. *The Review of Economics and Statistics* 2019;101:341–54.


For peer review only - http://bmjopen.bmj.com/site/about/guidelines.xhtml

28. Hanson M, Gluckman P. Developmental origins of noncommunicable disease: population and public health implications. *Am J Clin Nutr* 2011;94:1754S–1758S.

29. Chee Wai Ku, Rachael Si Xuan Loo, Cheryl Jia En Lim, *et al*. Development and Validation of a Lifestyle Behavior Tool in Overweight and Obese Women through Qualitative and Quantitative Approaches. *Nutrients* 2021, 13, 4553.

30. Ajzen I. The theory of planned behavior. *Organizational Behavior and Human Decision Processes* 1991;50:179–211.

31. Thaler RH, Sunstein CR. *Nudge: improving decisions about health. Wealth, and Happiness*. Yale University Press, 2008.

32. Shilts MK, Horowitz M, Townsend MS. Goal setting as a strategy for dietary and physical activity behavior change: a review of the literature. *Am J Health Promot* 2004;19:81–93.

33. Armitage CJ, Conner M. Efficacy of the Theory of Planned Behaviour: a meta-analytic review. *Br J Soc Psychol* 2001;40:471–99.

34. Cox JL, Holden JM, Sagovsky R. Detection of postnatal depression. Development of the 10-item Edinburgh Postnatal Depression Scale. *Br J Psychiatry* 1987;150:782–86.

35. Rosen R, Brown C, Heiman J, *et al*. The Female Sexual Function Index (FSFI): a multidimensional self-report instrument for the assessment of female sexual function. *J Sex Marital Ther* 2000;26:191–208.

36. Lim SX, Colega MT, M Ayob MN, *et al*. Identification and reproducibility of dietary patterns assessed with a FFQ among women planning pregnancy. *Public Health Nutr* 2021;24:2437–46.

37. Stunkard AJ, Messick S. The three-factor eating questionnaire to measure dietary restraint, disinhibition and hunger. *J Psychosom Res* 1985;29:71–83.


For peer review only



For peer review only - http://bmjopen.bmj.com/site/about/guidelines.xhtml

38. Craig CL, Marshall AL, Sjöström M, *et al*. International physical activity questionnaire: 12-country reliability and validity. *Med Sci Sports Exerc* 2003;35:1381–95.

39. IPAQ research committee. Guidelines for data processing and analysis of the international physical activity questionnaire-short and long forms, 2004.

40. Padmapriya N, Shen L, Soh SE, *et al*. Physical Activity and Sedentary Behavior Patterns Before and During Pregnancy in a Multi-ethnic Sample of Asian Women in Singapore. *Matern Child Health J* 2015;19:2523–35.

41. Buysse DJ, Reynolds CF, Monk TH, *et al*. The Pittsburgh Sleep Quality Index: a new instrument for psychiatric practice and research. *Psychiatry Res* 1989;28:193–213.

42. Ku CW, Leow SH, Ong LS, *et al*. Development of a Lifestyle Intervention Program for Overweight or Obese Preconception, Pregnant and Postnatal Women: a Qualitative Study. PREPRINT (version 1) *Research Square* 2021. https://doi.org/10.21203/rs.3.rs-877807/v1

43. Spira A. The use of fecundability in epidemiological surveys. *Hum Reprod* 1998;13:1753–6.

44. Wilcox AJ. *Fertility and pregnancy: an epidemiologic perspective*. United Kingdom: Oxford University Press, 2010.

45. Loo EXL, Soh SE, Loy S, *et al*. Cohort profile: Singapore Preconception Study of Long-Term Maternal and Child Outcomes (S-PRESTO). *Eur J Epidemiol* 2021;36:129–42.

46. Royston P. Multiple Imputation of Missing Values. *Stata J* 2004;4:227–41.

47. Cheung YB. Analysis of repeated measurements and clustered data. In: *Statistical analysis of human growth and development*. USA, Boca Raton (FL): CRC Press, 2014.

48. Rubin DB. *Multiple imputation for nonresponse in surveys*. USA, New York: John Wiley & Sons, 2004.

49. Personal Communications, Loy See Ling/Fabian Yap/Jerry Chan et al in Submission



For peer review only - http://bmjopen.bmj.com/site/about/guidelines.xhtml

50. Marchi J, Berg M, Dencker A, *et al*. Risks associated with obesity in pregnancy, for the mother and baby: a systematic review of reviews. *Obes Rev* 2015;16:621-38.

51. Dachew BA, Ayano G, Betts K, *et al*. The impact of pre-pregnancy BMI on maternal depressive and anxiety symptoms during pregnancy and the postpartum period: A systematic review and meta-analysis. *J Affect Disord* 2021;281:321-30.

52. Catalano PM, Shankar K. Obesity and pregnancy: mechanisms of short term and long term adverse consequences for mother and child. *BMJ* 2017;356:j1.

53. Soh SE, Tint MT, Gluckman PD, *et al*. Cohort profile: Growing Up in Singapore Towards healthy Outcomes (GUSTO) birth cohort study. *Int J Epidemiol* 2014;43:1401–09.

54. Burgess E, Hassmén P, Pumpa KL. Determinants of adherence to lifestyle intervention in adults with obesity: a systematic review. *Clin Obes* 2017;7:123-35.

For peer review only

## A continuum of care framework in Healthy Early Life Moments in Singapore

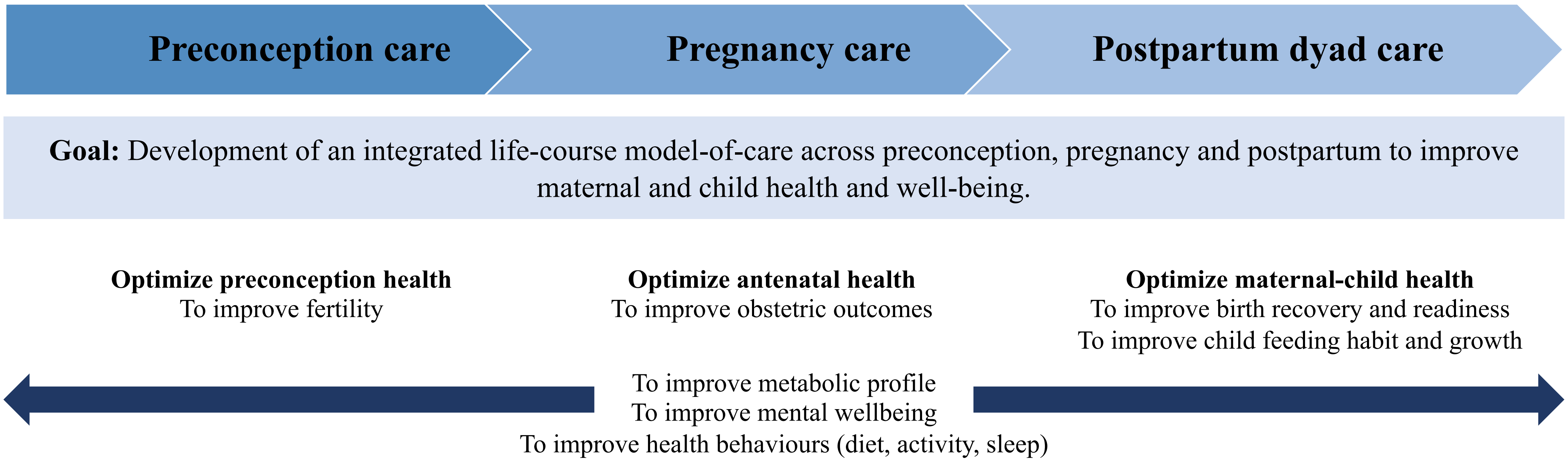

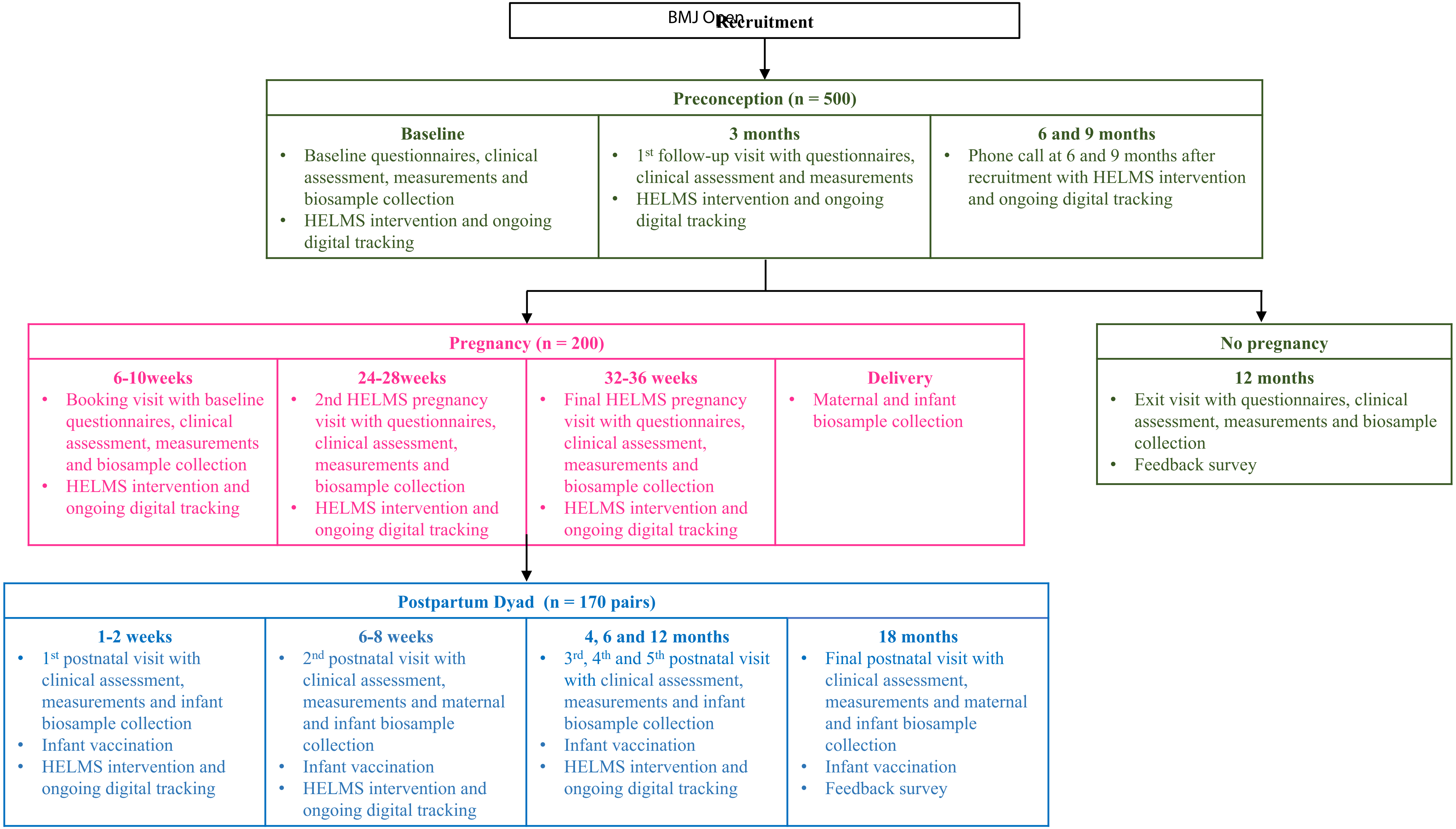
Recruitment
Preconception (n = 500)
Baseline
Baseline questionnaires, clinical assessment, measurements and biosample collection
HELMS intervention and ongoing digital tracking
3 months
1st follow-up visit with questionnaires, clinical assessment and measurements
HELMS intervention and ongoing digital tracking
6 and 9 months
Phone call at 6 and 9 months after recruitment with HELMS intervention and ongoing digital tracking
Pregnancy (n = 200)
6-10weeks
Booking visit with baseline questionnaires, clinical assessment, measurements and biosample collection
HELMS intervention and ongoing digital tracking
24-28weeks
2nd HELMS pregnancy visit with questionnaires, clinical assessment, measurements and biosample collection
HELMS intervention and ongoing digital tracking
32-36 weeks
Final HELMS pregnancy visit with questionnaires, clinical assessment, measurements and biosample collection
HELMS intervention and ongoing digital tracking
Delivery
Maternal and infant biosample collection
No pregnancy
12 months
Exit visit with questionnaires, clinical assessment, measurements and biosample collection
Feedback survey
Postpartum Dyad (n = 170 pairs)
1-2 weeks
1st postnatal visit with clinical assessment, measurements and infant biosample collection
Infant vaccination
HELMS intervention and ongoing digital tracking
6-8 weeks
2nd postnatal visit with clinical assessment, measurements and maternal and infant biosample collection
Infant vaccination
HELMS intervention and ongoing digital tracking
4, 6 and 12 months
3rd, 4th and 5th postnatal visit with clinical assessment, measurements and infant biosample collection
Infant vaccination
HELMS intervention and ongoing digital tracking
18 months
Final postnatal visit with clinical assessment, measurements and maternal and infant biosample collection
Infant vaccination
Feedback survey

STANDARD PROTOCOL ITEMS: RECOMMENDATIONS FOR INTERVENTIONAL TRIALS

SPIRIT 2013 Checklist: Recommended items to address in a clinical trial protocol and related documents*

| Section/item | Item No | Description | Addressed on page number |
|---|---|---|---|
| **Administrative information** | | | |
| Title | 1 | Descriptive title identifying the study design, population, interventions, and, if applicable, trial acronym | ______1_______ |
| Trial registration | 2a | Trial identifier and registry name. If not yet registered, name of intended registry | ______4______ |
| | 2b | All items from the World Health Organization Trial Registration Data Set | ______4_______ |
| Protocol version | 3 | Date and version identifier | ______23______ |
| Funding | 4 | Sources and types of financial, material, and other support | ______24_____ |
| Roles and responsibilities | 5a | Names, affiliations, and roles of protocol contributors | ______23_____ |
| | 5b | Name and contact information for the trial sponsor | ______24_____ |
| | 5c | Role of study sponsor and funders, if any, in study design; collection, management, analysis, and interpretation of data; writing of the report; and the decision to submit the report for publication, including whether they will have ultimate authority over any of these activities | ______23_____ |
| | 5d | Composition, roles, and responsibilities of the coordinating centre, steering committee, endpoint adjudication committee, data management team, and other individuals or groups overseeing the trial, if applicable (see Item 21a for data monitoring committee) | ______22_______ |

For peer review only

**Introduction**

| | | | |
|---|---|---|---|
| Background and rationale | 6a | Description of research question and justification for undertaking the trial, including summary of relevant studies (published and unpublished) examining benefits and harms for each intervention | ______5-7____ |
| | 6b | Explanation for choice of comparators | ______5-7____ |
| Objectives | 7 | Specific objectives or hypotheses | ______7_______ |
| Trial design | 8 | Description of trial design including type of trial (eg, parallel group, crossover, factorial, single group), allocation ratio, and framework (eg, superiority, equivalence, noninferiority, exploratory) | ______8-9_____ |

**Methods: Participants, interventions, and outcomes**

| | | | |
|---|---|---|---|
| Study setting | 9 | Description of study settings (eg, community clinic, academic hospital) and list of countries where data will be collected. Reference to where list of study sites can be obtained | ______8-9_____ |
| Eligibility criteria | 10 | Inclusion and exclusion criteria for participants. If applicable, eligibility criteria for study centres and individuals who will perform the interventions (eg, surgeons, psychotherapists) | ______10_____ |
| Interventions | 11a | Interventions for each group with sufficient detail to allow replication, including how and when they will be administered | ______11-14__ |
| | 11b | Criteria for discontinuing or modifying allocated interventions for a given trial participant (eg, drug dose change in response to harms, participant request, or improving/worsening disease) | ______10______ |
| | 11c | Strategies to improve adherence to intervention protocols, and any procedures for monitoring adherence (eg, drug tablet return, laboratory tests) | ______15_______ |
| | 11d | Relevant concomitant care and interventions that are permitted or prohibited during the trial | ______11-14____ |
| Outcomes | 12 | Primary, secondary, and other outcomes, including the specific measurement variable (eg, systolic blood pressure), analysis metric (eg, change from baseline, final value, time to event), method of aggregation (eg, median, proportion), and time point for each outcome. Explanation of the clinical relevance of chosen efficacy and harm outcomes is strongly recommended | ______15-17____ |
| Participant timeline | 13 | Time schedule of enrolment, interventions (including any run-ins and washouts), assessments, and visits for participants. A schematic diagram is highly recommended (see Figure) | _____Figure 2__ |



For peer review only - http://bmjopen.bmj.com/site/about/guidelines.xhtml

| | | | |
|---|---|---|---|
| Sample size | 14 | Estimated number of participants needed to achieve study objectives and how it was determined, including clinical and statistical assumptions supporting any sample size calculations | _____19________ |
| Recruitment | 15 | Strategies for achieving adequate participant enrolment to reach target sample size | _____10________ |

**Methods: Assignment of interventions (for controlled trials)**

Allocation:

| | | | |
|---|---|---|---|
| Sequence generation | 16a | Method of generating the allocation sequence (eg, computer-generated random numbers), and list of any factors for stratification. To reduce predictability of a random sequence, details of any planned restriction (eg, blocking) should be provided in a separate document that is unavailable to those who enrol participants or assign interventions | ______NA______ |
| Allocation concealment mechanism | 16b | Mechanism of implementing the allocation sequence (eg, central telephone; sequentially numbered, opaque, sealed envelopes), describing any steps to conceal the sequence until interventions are assigned | ______NA______ |
| Implementation | 16c | Who will generate the allocation sequence, who will enrol participants, and who will assign participants to interventions | ______NA______ |
| Blinding (masking) | 17a | Who will be blinded after assignment to interventions (eg, trial participants, care providers, outcome assessors, data analysts), and how | ______NA______ |
| | 17b | If blinded, circumstances under which unblinding is permissible, and procedure for revealing a participant's allocated intervention during the trial | ______NA______ |

**Methods: Data collection, management, and analysis**

| | | | |
|---|---|---|---|
| Data collection methods | 18a | Plans for assessment and collection of outcome, baseline, and other trial data, including any related processes to promote data quality (eg, duplicate measurements, training of assessors) and a description of study instruments (eg, questionnaires, laboratory tests) along with their reliability and validity, if known. Reference to where data collection forms can be found, if not in the protocol | _____15-17_____ |
| | 18b | Plans to promote participant retention and complete follow-up, including list of any outcome data to be collected for participants who discontinue or deviate from intervention protocols | ______15-17___ |

| | | | |
|---|---|---|---|
| Data management | 19 | Plans for data entry, coding, security, and storage, including any related processes to promote data quality (eg, double data entry; range checks for data values). Reference to where details of data management procedures can be found, if not in the protocol | _____22-23____ |
| Statistical methods | 20a | Statistical methods for analysing primary and secondary outcomes. Reference to where other details of the statistical analysis plan can be found, if not in the protocol | ____18_____ |
| | 20b | Methods for any additional analyses (eg, subgroup and adjusted analyses) | ____18____ |
| | 20c | Definition of analysis population relating to protocol non-adherence (eg, as randomised analysis), and any statistical methods to handle missing data (eg, multiple imputation) | _____18____ |
| **Methods: Monitoring** | | | |
| Data monitoring | 21a | Composition of data monitoring committee (DMC); summary of its role and reporting structure; statement of whether it is independent from the sponsor and competing interests; and reference to where further details about its charter can be found, if not in the protocol. Alternatively, an explanation of why a DMC is not needed | _____23________ |
| | 21b | Description of any interim analyses and stopping guidelines, including who will have access to these interim results and make the final decision to terminate the trial | ______NA______ |
| Harms | 22 | Plans for collecting, assessing, reporting, and managing solicited and spontaneously reported adverse events and other unintended effects of trial interventions or trial conduct | ______NA______ |
| Auditing | 23 | Frequency and procedures for auditing trial conduct, if any, and whether the process will be independent from investigators and the sponsor | ______22_______ |
| **Ethics and dissemination** | | | |
| Research ethics approval | 24 | Plans for seeking research ethics committee/institutional review board (REC/IRB) approval | _____22______ |
| Protocol amendments | 25 | Plans for communicating important protocol modifications (eg, changes to eligibility criteria, outcomes, analyses) to relevant parties (eg, investigators, REC/IRBs, trial participants, trial registries, journals, regulators) | _____NA_______ |



For peer review only - http://bmjopen.bmj.com/site/about/guidelines.xhtml

| | | | |
|---|---|---|---|
| Consent or assent | 26a | Who will obtain informed consent or assent from potential trial participants or authorised surrogates, and how (see Item 32) | ______8, 22______ |
| | 26b | Additional consent provisions for collection and use of participant data and biological specimens in ancillary studies, if applicable | _______NA______ |
| Confidentiality | 27 | How personal information about potential and enrolled participants will be collected, shared, and maintained in order to protect confidentiality before, during, and after the trial | _______23________ |
| Declaration of interests | 28 | Financial and other competing interests for principal investigators for the overall trial and each study site | _______24________ |
| Access to data | 29 | Statement of who will have access to the final trial dataset, and disclosure of contractual agreements that limit such access for investigators | ______NA________ |
| Ancillary and post-trial care | 30 | Provisions, if any, for ancillary and post-trial care, and for compensation to those who suffer harm from trial participation | _______NA_______ |
| Dissemination policy | 31a | Plans for investigators and sponsor to communicate trial results to participants, healthcare professionals, the public, and other relevant groups (eg, via publication, reporting in results databases, or other data sharing arrangements), including any publication restrictions | _______NA_______ |
| | 31b | Authorship eligibility guidelines and any intended use of professional writers | ______NA________ |
| | 31c | Plans, if any, for granting public access to the full protocol, participant-level dataset, and statistical code | ______NA________ |
| **Appendices** | | | |
| Informed consent materials | 32 | Model consent form and other related documentation given to participants and authorised surrogates | ______NA________ |
| Biological specimens | 33 | Plans for collection, laboratory evaluation, and storage of biological specimens for genetic or molecular analysis in the current trial and for future use in ancillary studies, if applicable | ______17_________ |

*It is strongly recommended that this checklist be read in conjunction with the SPIRIT 2013 Explanation & Elaboration for important clarification on the items. Amendments to the protocol should be tracked and dated. The SPIRIT checklist is copyrighted by the SPIRIT Group under the Creative Commons "Attribution-NonCommercial-NoDerivs 3.0 Unported" license.

BMJ Open

# Effects of an integrated mobile health lifestyle intervention among overweight and obese women planning for pregnancy in Singapore: protocol for the single-arm Healthy Early Life Moments in Singapore (HELMS) study

| | |
|---|---|
| Journal: | *BMJ Open* |
| Manuscript ID | bmjopen-2022-061556.R1 |
| Article Type: | Protocol |
| Date Submitted by the Author: | 28-Oct-2022 |
| Complete List of Authors: | Chan, Jerry; KK Women's and Children's Hospital, Department of Reproductive Medicine<br>Ku, Chee Wai; KK Women's and Children's Hospital, Department of Reproductive Medicine; Duke-NUS Medical School,<br>Loy, See Ling; KK Women's and Children's Hospital, Department of Reproductive Medicine; Duke-NUS Medical School<br>Godfrey, Keith; University of Southampton, MRC Lifecourse Epidemiology Unit; University of Southampton, National Institute for Health Research Southampton Biomedical Research Centre<br>Fan, Yiping; KK Women’s and Children’s Hospital, Department of Reproductive Medicine<br>Chua, Mei Chien; Duke-NUS Medical School; KK Women’s and Children’s Hospital, Department of Neonatology<br>Yap, Fabian; KK Women's and Children's Hospital, Department of Paediatrics; NUS Yong Loo Lin School of Medicine |
| <b>Primary Subject Heading</b>: | Obstetrics and gynaecology |
| Secondary Subject Heading: | Reproductive medicine, Public health, Nutrition and metabolism |
| Keywords: | MENTAL HEALTH, Other metabolic, e.g. iron, porphyria < DIABETES & ENDOCRINOLOGY, NUTRITION & DIETETICS |
| | |

SCHOLARONE™
Manuscripts

# Effects of an integrated mobile health lifestyle intervention among overweight and obese women planning for pregnancy in Singapore: protocol for the single-arm Healthy Early Life Moments in Singapore (HELMS) study

Jerry Kok Yen Chan[1,2,3], Chee Wai Ku[1,2], See Ling Loy[1,2], Keith M. Godfrey[4,5], Yiping Fan[1], Mei Chien Chua[2,6], Fabian Yap[2,7,8]

[1]Department of Reproductive Medicine, KK Women's and Children's Hospital, Singapore, Singapore 229899

[2]Duke-NUS Medical School, Singapore, Singapore 169857

[3]Yong Loo Lin School of Medicine, National University of Singapore, Singapore, Singapore 119228

[4]Medical Research Council Lifecourse Epidemiology Centre, University of Southampton, Southampton SO16 6YD, United Kingdom

[5]National Institute for Health Research Southampton Biomedical Research Centre, University of Southampton and University Hospital Southampton National Health Service Foundation Trust, Southampton SO16 6YD, United Kingdom

[6]Department of Neonatology, KK Women's and Children's Hospital, Singapore, Singapore 229899

[7]Department of Paediatrics, KK Women's and Children's Hospital, Singapore, Singapore 229899

[8]Lee Kong Chian School of Medicine, Nanyang Technological University, Singapore, Singapore 636921

Corresponding Authors: Jerry Kok Yen Chan and Fabian Yap

For peer review only

Department of Reproductive Medicine, KK Women's and Children's Hospital, Singapore, Singapore 229899

jerrychan@duke-nus.edu.sg

Department of Paediatrics, KK Women's and Children's Hospital, Singapore, Singapore 229899

fabian.yap.k.p@singhealth.com.sg

Word count: 4794

Keywords: Obesity, metabolic health, mental health, lifestyle intervention, preconception

For peer review only

## ABSTRACT

### Introduction

Changes in social and lifestyle factors have led to increasing rates of metabolic and mental health problems. We hypothesize that a transformation of the current maternal and child health system is required to deliver interventions that effectively promote a good start to life in populations at risk of metabolic and mental health problems. We describe a single-arm implementation study "Healthy Early Life Moments in Singapore" (HELMS), which aims to examine whether an integrated lifestyle intervention initiated at preconception and continuing throughout pregnancy and postpartum periods can improve the metabolic and mental health of overweight and obese women, and improve early child growth.

### Methods and Analysis

This single centre implementation trial is conducted at KK Women's and Children's Hospital (KKH), Singapore. The trial aims to recruit 500 women, aged 21-40 years with a body mass index of 25-40 kg/m$^2$ who plan to get pregnant, with interventions delivered before conception, until 18 months post-delivery. Primary outcomes comprise pregnancy rate, maternal metabolic and mental health status. Secondary outcomes include maternal reproductive health, pregnancy outcomes, and offspring growth. The intervention will be delivered using a mobile health application, to provide anticipatory guidance, raise awareness, and guide goal-setting on lifestyle behaviours that include diet, physical activity, mental wellness, and sleep hygiene from preconception to postpartum. Women who conceive within one year of recruitment will be followed through pregnancy and studied with their infants at six time points during the first 18 months of life. Questionnaires, anthropometric measurements and multiple biosamples will be collected at each visit.

### Ethics and Dissemination


For peer review only

The study has been approved by the Centralised Institutional Review Board of SingHealth (2021/2247). Written informed consent will be obtained from all participants. The findings will be published in peer-reviewed journals and disseminated to national and international policy makers.

**Trial registration**

ClinicalTrials.gov (NCT05207059).

**ARTICLE SUMMARY**

**Strengths and limitations of this study**

- HELMS aims to examine the impact of integrated interventions, initiated preconceptionally and continued throughout the pregnancy and postpartum phases, on metabolic and mental health of women who are overweight or obese.
- Extensive biosampling and detailed phenotyping of mother, father, and child will allow longitudinal assessment of changes in health behaviours including diet, physical activity and sleep, maternal metabolic profile, and depression risk.
- This will provide an important platform for biomarker discovery, validate integrated HELMS interventions, and pave the way for new guidelines for lifestyle programs from preconception to postpartum stages.
- The main limitation of this study design includes the inability to distinguish between the effect of intervention, a placebo effect, and the effect of natural history.
- HELMS is a single-centre study of the Asian population, involving only English-speaking participants, so external validity may be limited to this population, and caution should be exercised in generalizing results across different settings and populations.

For peer review only

## INTRODUCTION

Non-communicable diseases (NCDs) represent the predominant cause of morbidity and mortality worldwide.[1] Rapid urbanization, unhealthy diet and sedentary lifestyle have led to an epidemic of metabolic diseases, which are the main drivers of NCDs.[1] Coupled with this metabolic epidemic is a rising rate of mental disorders, especially depression, as the leading cause of disability worldwide that commonly affects women.[2] Both metabolic and mental disorders are interrelated, with their co-occurrence frequently observed in individuals living with obesity.[3 4] The short- and long-term health risks of maternal obesity and depression in mothers and their children are well documented.[5–7] Women living with obesity and mental health conditions are at increased risk of infertility, adverse obstetric outcomes, and postpartum cardiometabolic complications; and their children are also susceptible to obesity, metabolic disease, and psychosocial disturbances in childhood and adolescence.[5–7] In Singapore, almost one-third of women are overweight or obese [body mass index (BMI) 25-29 kg/m$^2$ :17%; BMI ≥30 kg/m$^2$ :13%] before pregnancy,[8] while one in ten women experience depression before, during and after pregnancy.[9 10]

To mitigate the impact of maternal obesity and depression, various intervention strategies targeting at antenatal and postpartum periods have been studied. These include setting SMART (Specific, Measurable, Achievable, Relevant, Time-Bound) goals, providing healthy lifestyle counselling or group-based education, perinatal mental health interventions, and providing health service support.[11–13] However, these antenatal- and immediate postpartum-phase focused interventions have had modest success,[14 15] only some showing improvements in eating behaviour, physical activity or weight status,[11 16–18] and most failing to prevent adverse mother-offspring outcomes such as gestational diabetes mellitus (GDM) and macrosomia. This could be attributed to the relatively short duration of the intervention, the

For peer review only



For peer review only - http://bmjopen.bmj.com/site/about/guidelines.xhtml

lack of continued care from preconception, and intervention only beginning in the antenatal and postpartum periods, which may be too late to produce any meaningful impact.

There is increasing focus on interventions during the preconception period, especially for women living with obesity, who are susceptible to both metabolic and mental disorders.[3 19] Such interventions not only improve the health of the mother, but also provide potential health benefits to the next generation through an improved environment for embryo and foetal development.[20 21] The preconception period represents a unique opportunity where women are motivated to make a positive change to attain optimal pregnancy outcomes.[22] So far, preconception lifestyle interventions have demonstrated positive maternal behaviour changes, such as increased intake of multivitamins, vegetables and other dietary changes, increased physical activity, as well as smoking cessation and reduced alcohol consumption, resulting in reduction in BMI and gestational weight gain (GWG), higher clinical pregnancy rate and lower preterm delivery.[23–25] This has led to improved knowledge, self-efficacy, and control.[26] Furthermore, women who achieved at least 5% weight loss during such interventions had better cardiometabolic health based on glycaemic and lipid measures six years later.[25]

Taken together, this suggests that adopting a life-course perspective in the healthcare model with a continuum of care provided before, during, and after pregnancy has the potential to address the trajectory of increased metabolic and mental disorder risk throughout these life stages. Acting upstream before conception to optimize health is crucial to improving reproductive potential while reducing the social and health implications resulting from unplanned pregnancies. Modifying current antenatal care services by empowering women in preparation for pregnancy and childbirth is critical for the wellbeing of both mother and baby.



For peer review only - http://bmjopen.bmj.com/site/about/guidelines.xhtml

Optimizing postpartum recovery and readiness, and nurturing infants through optimal feeding practices and growth monitoring are important for promoting virtuous life cycles of health in an individual and breaking vicious life cycles of NCDs for the next generation.[27]

**GOALS AND AIMS**

We envision an integrated life-course approach with a continuum of care that encompasses preconception preparation, pregnancy optimization, along with postpartum synchronization of maternal-child health services in the first 18 months of life. Connecting the preconception, pregnancy and postpartum journeys will achieve synergism in producing greater behavioural change and beneficial outcomes not only for women, but also for the child and family.[19] Therefore, the goal of Healthy Early Life Moments in Singapore (HELMS) is to develop and implement a life-course model of care (MOC) starting from preconception to pregnancy and postpartum phases, to achieve optimal metabolic and mental health for both mother and child. This MOC focuses on preventive healthcare, a time where the cost effectiveness of intervention is likely to be maximum,[28] and represents the clinical translation of early developmental programming based on the Developmental Origins of Health and Disease (DOHaD) paradigm.[20]

In this study, the overarching aim is to examine whether an integrated intervention beginning preconceptionally and continuing throughout the pregnancy and postpartum phases can improve the metabolic and mental health of women living with obesity, as well as optimise offspring growth and development. At each life-course phase, the study addresses specific hypotheses. We hypothesize that the HELMS MOC lifestyle interventions will promote metabolic and mental health of overweight and obese women, and thus optimize (i) reproductive outcomes during preconception, (ii) obstetric outcomes during pregnancy, and

For peer review only

(iii) postpartum physical and mental wellbeing, and healthy feeding habits and growth during infancy. We also hypothesize that greater improvements in metabolic and mental health will be observed in obese women with obesity than those who are overweight. An overview of the study framework is shown in **Figure 1**.

## METHODS AND ANALYSIS

### Trial Design and Setting

This study represents a model of implementation to demonstrate the value of adopting a life-course approach and integrated care to maternal-child health. It is conceptualized as a single-arm trial, with a targeted intervention implemented in a group of women living with obesity who are trying to conceive within one year after recruitment. Longitudinal intervention will be performed from preconception, throughout pregnancy until 18 months postpartum in the KK Women's and Children's Hospital (KKH), Singapore. The protocol is written in accordance with the SPIRIT guidelines. Written informed consent will be obtained from all participants at the recruitment.

The flow of the trial is shown in **Figure 2**. Participants will be reviewed at multiple time points from preconception through pregnancy, and both participants and their infants will be followed for the first 18 months after delivery, with extensive biosampling and detailed phenotyping performed longitudinally. Following informed consent at the first preconception clinic visit, participants will complete a set of questionnaires that assess baseline demographics, diet practices, physical activity, sleep, emotion, and sexual health. Anthropometric measurements will be taken, together with biosample collection, including blood for a standard 75g oral glucose tolerance test (OGTT) and stool. Participants will receive a doctor's consultation, along with prescription of a standard preconception



For peer review only - http://bmjopen.bmj.com/site/about/guidelines.xhtml

supplement, digitized preconception care and healthy lifestyle guidance. The second preconception clinic visit will take place three months later, with questionnaires and measurements being performed, and compliance to the intervention being reviewed. Phone contact will be made six and nine months after recruitment to help participants maintain focus on their preconception health and lifestyle goals, and to remind them to perform a urine pregnancy test if periods are missed. If they do not become pregnant, an exit visit will be conducted at 12 months with questionnaires, measurements, and biosampling, followed by appropriate evaluations and subfertility referrals thereafter. For participants who become pregnant, they will be seen between 6-10 weeks of gestation at the first pregnancy visit. Questionnaires, measurements, and biosampling will again be performed. The second HELMS pregnancy visit will be at 24-28 weeks, and the final HELMS visit in pregnancy will be at 32-36 weeks. Continuous antenatal care and healthy lifestyle guidance during pregnancy will be provided throughout the trimesters. At delivery, maternal and infant biosampling, including placenta collection, will be performed. In the postpartum dyad phase, there will be 6 visits over an 18-month period, according to the infant vaccination schedule, at 1-2 weeks, 6-8 weeks, 4, 6, 12, and 18 months. An ongoing intervention tailored to this postpartum dyad phase will be delivered, together with questionnaires, measurements, and biosampling for mother and child at each visit. Throughout the study, participants will be asked to log their weight and supplement intake weekly in a digital calendar. For the partner, lifestyle and anthropometric measurements will be collected during the preconception, pregnancy, and postpartum periods; blood will be collected once during the pregnancy phase. Meanwhile, partners will be passively engaged in the intervention by receiving the same set of digitized intervention materials. A feedback survey on the HELMS program will be performed at the end of each preconception, pregnancy, and postpartum phases.

For peer review only



For peer review only - http://bmjopen.bmj.com/site/about/guidelines.xhtml

**Patients and public involvement**

We have conducted in-depth face-to-face interviews with preconception, pregnant and postpartum women who were overweight and obese to understand their needs throughout the preconception-pregnancy-postpartum journey, enablers and barriers to a healthy lifestyle, intervention preferences, and delivery methods. This work enabled development of the intervention package and dissemination plans.[29]

**Participants and recruitment**

Potential participants from the public or KKH (patients/ staff) or referred from other healthcare institutes who fulfil the inclusion criteria will be invited to participate in the HELMS study. KKH houses the largest Obstetrics and Gynaecology department in Singapore, managing approximately 12,000 deliveries a year with patients made up of a wide sociodemographic spectrum. Invitation letters, emails, messages, webpages, brochures, and posters will be used for advertising. Interested women can contact the study team by email or phone.

Inclusion criteria include (1) woman who plans to conceive in the next 12 months; (2) BMI 25-40 kg/m$^2$; (3) age 21-40 years; (4) Chinese, Malay, Indian ethnicity, or any combination of these three ethnic groups; (5) intending to reside in Singapore for the next four years; (5) can access the Internet through any digital platform; and (6) can provide written informed consent. Exclusion criteria include (1) currently pregnant; (2) difficulty in understanding the English language; (3) known Type 1 or Type 2 diabetes; (4) on anticonvulsant medication, oral steroid, contraception, or fertility medication in the past one month; (5) on HIV or Hepatitis B or C medication in the past one month. If a woman is pregnant within one month from the baseline visit, they will be censored from the analyses. If a woman has a pregnancy

For peer review only



For peer review only - http://bmjopen.bmj.com/site/about/guidelines.xhtml

loss and wishes to re-join the study, she will be re-characterized as at the preconception baseline visit at least one month after a negative urinary pregnancy test and will receive intervention as before.

Women who suffer from a miscarriage or termination event, experience multiple pregnancies, with foetal congenital anomalies, cannot comply with the study protocol or wish to discontinue their participation will be withdrawn from the study.

For peer review only

**Intervention overview**

All participants will receive an intervention via a mobile health application, designed to address and improve women's knowledge, attitude, and practice in terms of preconception-pregnancy-postpartum care and health behaviours. There will be four modules, namely the HELMS Journey, the HELMS Model, the HELMS Lifestyle, and the HELMS Community. The HELMS Journey will provide anticipatory guidance on examinations and measurements performed during clinic visits. The HELMS Model will deliver the 4S (Screening, Size, Supplementation, Special considerations) care plans. The HELMS Lifestyle will provide support in healthy eating, physical activity, mental wellness, and sleep hygiene. Finally, the HELMS Community will provide social support and improve participation in the program. Each of these modules contains phase-specific information developed by obstetricians, neonatologists, paediatricians, dietitians, physiotherapists, psychiatrists, and psychologists.

**Intervention Details**

*1. HELMS Journey*



For peer review only - http://bmjopen.bmj.com/site/about/guidelines.xhtml

Details of each preconception, pregnancy, and postpartum visit of HELMS will be available here to provide anticipatory guidance on these visits. This will ensure that participants are aware of the study measurements, as well as the required routine examinations.

*2. HELMS Model*

The HELMS life-course interventions, namely 4S, are developed to provide care throughout the journey from preconception to postnatal. 4S is represented by '**S**creening', '**S**ize', '**S**upplementation' and '**S**pecial considerations'.

- **Screening** involves health and risk assessments through physical and biomarker (e.g. Anti-Mullerian hormone, glucose, insulin and lipid profile) measurements, as well as emotion and sleep evaluations for mothers, and developmental assessment for children.
- Body **Size** management encompasses education on weight status awareness and mother-child health implications, both mother-child weight tracking, healthy eating, and physical activity guidance.
- **Supplementation** includes multi-micronutrient, vitamin D, calcium, and / or DHA supplements, which are phase specific for mother, and vitamin D supplement drops for the child.
- **Special considerations** include the management of preconception sexual health and function, the management of pregnancy symptoms and postpartum recovery, and the monitoring of infant growth and feeding management.

*3. HELMS Lifestyle*

Metabolic health support (Diet and Physical activity modules)

For peer review only

The **diet module** is developed based on the 6P tool, which is designed as a platform to address metabolic health based on the principles of energy input and expenditure, as well as motivation as the basis for dietary change.[30] It is a simple, self-administered instrument based on the concept of a mental model, which can lead to self-awareness, self-evaluation, and self-education, over time resulting in a positive change in eating habits and health. The 6P comprises 6 components as presented in **Table 1**. Multiple modalities are incorporated that include feedback on 6P behaviour, 6P self-monitoring, 6P goal setting, and 6P nudges along with the use of the 6P tool. By including these 6 main discrete dietary and activity components under a single 'package', it will provide an overview of the mental model of nutrition based on the principle of energy input and expenditure, promoting self-awareness of unhealthy lifestyle behaviours and nudging individuals into actual implementation via concrete, personalized feedback and increasing intrinsic motivation. This is based on the theoretical framework of the Theory of Planned Behaviour (TPB) which has been widely used to predict behavioural intention and health-related behaviors.[31]

**Table 1** Components of the 6P tool

| 6P | Healthy mental model of nutrition | Description |
|---|---|---|
| P1 | PORTION | Amount of food intake |
| P2 | PROPORTION | Caloric density of food intake |
| P3 | PLEASURE | Frequency of snacks and sugary beverages and irregular intake |
| P4 | PERIOD | Time of day of food intake |
| P5 | PHYSICALITY | Physical activity and sedentary behaviour |
| P6 | PSYCHOLOGY | Readiness for change |

For peer review only

The **physical activity module** seeks to engage women with the importance, type, and intensity of exercise during each phase. It consists of a series of appropriate, tailored exercise videos developed by physiotherapists and obstetricians with the support of scientific evidence. Wearable activity trackers will be used to provide real-time feedback on physical performance and help participants monitor their energy expenditure. Sustainability will be supported with the use of nudges (both text messages and images) delivered weekly to influence behaviour and decision-making.

Mental health support (Mental health and Sleep modules)

The **mental health module** serves as the main way to support, screen, and guide women's mental health longitudinally. Upstream screening of depressive symptoms at the preconception stage allows women at high risk to be triaged earlier for receiving targeted mental health support from KKH's mental health team comprising a clinical coordinator, case manager and psychiatrist. Women at medium risk will be engaged more intensively, while those at low risk will continue to participate, enabling and preparing for different stages through a package of clinical care and lifestyle support, especially in the late stages of pregnancy and early stages of postpartum. The **sleep module** aims to support mental wellness by promoting healthy sleep practices throughout the life-course. A wearable sleep tracker will be used to provide real-time feedback on sleep performance and help participants to monitor their sleep patterns.

*4. HELMS Community*

For peer review only

An online community platform is being established as part of supporting mental health among HELMS participants. This allows women to share their experience, identify common problems among peers and generate solutions that work for them in the online chat group.

Our intervention approach will be based on the **SIGN strategy** (Support, Inform, Guide and Nudge) to deliver the 4S and healthy lifestyle support. Close monitoring of women's health allows timely **support** provided by the healthcare staff. Setting up mobile health education helps to **inform** women about the importance and warning points in different stages. Educational materials in various formats, including diagrams and videos, provide **guidance** on phase-specific care and healthy lifestyles that focus on nutrition, physical activity, mental health, and sleep hygiene. Health **nudges** in the form of key messages related to preconception, pregnancy, and postpartum dyad care, as well as healthy lifestyles, facilitate care support and behavioural changes. The use of nudges represents a preferred architecture strategy that is widely used in public policy making, to alter people's behaviour and influence decision making.[32]

In summary, we will apply an interactive and personalized approach to conducting the intervention, supported by real-time feedback. This involves goal setting, individual education, self-awareness, self-monitoring, motivation, and outcome review process. These techniques are commonly used in behavioural interventions and can improve health outcomes.[33 34]

**Intervention adherence**

Adherence to the intervention will be established throughout the study with regular logbook charts of supplement intake, sexual activity, and weight, which will be reviewed at each study



For peer review only - http://bmjopen.bmj.com/site/about/guidelines.xhtml

visit or by telephone reminders. The use of a digital tracker for activity and sleep allows off-site monitoring of wearing adherence, and research staff will take action to check for reasons for non-adherence.

**Outcomes and assessments**

The primary outcome is the pregnancy rate in overweight and obese women trying to conceive within one year of baseline assessment. It is defined by a positive urinary pregnancy test, followed by ultrasound confirmation of an intrauterine gestational sac after six weeks of amenorrhea. If an ultrasound scan is not available or inconclusive, the diagnosis of pregnancy will be made clinically. A successful conception is one of the most important clinical outcomes, and it represents the culmination of improved metabolic and mental health, leading to optimized gametogenesis.

Co-primary outcomes include maternal metabolic health and mental health status in each phase, with 18 months post-delivery serving as the principal endpoint. Maternal metabolic health is assessed by metabolic syndrome criteria. Mental health is evaluated using the Edinburgh Postnatal Depression Scale (EPDS).[35]

Other key outcomes include:

**Women/mothers**

- Reproductive health as assessed by fecundability, pregnancy loss and live birth rates, and sexual function based on Female Sexual Function Index-19.[36]
- Pregnancy outcomes include pain, obstetric, and delivery complications.
- Health behaviours assessed by dietary practice (6P tool,[30] 4-day food diary, Food Frequency Questionnaire,[37] Three Factor Eating Questionnaire,[38] alcohol and



For peer review only - http://bmjopen.bmj.com/site/about/guidelines.xhtml

supplement intake), smoking exposure, physical activity, and sedentary behaviours (International Physical Activity Questionnaire,[39 40] Sedentary Behaviour Questionnaire,[41] accelerometer), and sleep (Pittsburgh Sleep Quality Index,[42] digital tracker).

- Metabolic profile, including weight status, body fat distribution, blood pressure, lipid, and glycaemic measures
- Gut microbiome profile assessed by stool sample.
- Nutrient status based on diet, blood, and breast milk composition (breast milk will be collected at five timepoints through the first year post-delivery).
- Ocular health as assessed by retinal vessel calibre characteristics

**Offspring**

- Anthropometry and growth assessed by antenatal serial ultrasound scans, infant weight, length, and head circumference.
- Neonatal complications such as hypoglycaemia and admission to neonatal care facilities; infant health and well-being, and skin biopsy for a subset of infants with eczema.
- Infant feeding assessed by breastfeeding behaviours, time of weaning, nutrition milestone, dietary intake and eating behaviours.

**Partners**

- Physical and metabolic health assessed by weight, BMI, and body fat distribution
- Health behaviours assessed by smoking exposure, alcohol intake, meal pattern, stress level, physical activity, and sedentary behaviours

For peer review only

Program effectiveness will be evaluated based on women's quality of life, healthcare utilization and participant feedback surveys. Blood, cord blood, stool, urine, saliva, placenta, and breast milk samples will be stored for analyses on biochemical, micronutrient, metabolomic, genomic, epigenetic, immunological, and molecular profiles.

**Planned analyses**

Continuous variables will be presented as means and standard deviations, or medians and 25th – 75th centiles, as appropriate. Categorical variables will be presented as numbers and percentages. The pregnancy rate will be determined by the number of women who became pregnant (defined by stated previously) divided by the total number of women who completed the 1-year follow-up during the preconception phase. The time to pregnancy (TTP) will be estimated by the number of menstrual cycles required to achieve a pregnancy over one year of follow-up. We will use the discrete-time proportional hazards model, which analyses TTP as a discrete scale based on the number of menstrual cycles, to estimate the fecundability ratio (FR) and the 95% confidence interval (CI),[43 44] accounting for left truncation and right censoring. For the other two co-primary outcomes, linear mixed effects model will be used to examine changes in means between the baseline and follow-up metabolic markers and EPDS scores, with adjustment for baseline potential confounders (including partner's characteristics) and duration of the intervention received. In addition to the pre-post comparison of outcomes stated above, differences with pregnancy and birth outcomes will also be compared with a similar observational cohort in Singapore called Singapore PREconception Study of long-Term maternal and child Outcomes (S-PRESTO),[45] using multiple linear or logistic regression models, adjusting for potential confounders. These confounders, such as age, ethnicity, and education, will be determined from literature

For peer review only

review, directed acyclic graph and/or observed statistically significant associations with exposures and outcomes.

We will impute missing data using multiple imputation analyses by chained equations.[46] The number of imputations will be determined based on percentage of missing values,[47] and results of total imputations will be pooled using Rubin's rule.[48] To determine whether imputation of the missing data may have affected the results, we will perform sensitivity analyses on participants with a complete set of data.

**Sample size calculation**

Based on the S-PRESTO study,[45] which is an observational cohort recruited from preconception and followed up through postpartum periods, preconception overweight or obese women (BMI ≥25 kg/m$^2$) had a 38% pregnancy rate, while those with normal weight (BMI 18.5 - 24.9) had 47% pregnancy rate. Among overweight and obese women, those with healthy metabolic profile characterised by absence of the metabolic syndrome or insulin resistance had a 52% pregnancy rate. We anticipated that HELMS intervention will improve overweight or obese women's pregnancy rate from 38% to 50% within 12 months of trying. Using a two-sided α level of 5% and a power of at least 90%, 400 participants are needed in the preconception phase, leading to 200 pregnancies. Considering a 20% dropout rate, 500 participants are required for recruitment. We estimated that 15% of women will experience pregnancy loss and another 10% dropout, leaving 150 dyad pairs to be followed.

**Quality control**

Procedures and actions will be implemented throughout the study to ensure that information provided to all participants is standardized, data collected is as complete as possible, and of high quality. Research staff responsible for recruiting and follow-up participants will receive

For peer review only


For peer review only - http://bmjopen.bmj.com/site/about/guidelines.xhtml

training from study leads on recruitment, consent taking, questionnaire and data management, intervention delivery and compliance monitoring. An operation database will be developed to monitor participant progression throughout the study, schedule study visits and monitor visit/measure completeness. A quarterly meeting will be held among study leads and research staff to review recruitment process, intervention delivery, data collection and participants' feedback. An annual audit on the study will be performed by an independent party.

**Data monitoring**

All data will be pseudonymised. Participants' identifiers will be kept separately in a password-protected file and only be assessed by specific research staff. Electronic data will be managed using a secure, encrypted online data capturing system approved by the institution. A data monitoring team will perform data checking on completeness, errors and outliers. To facilitate data monitoring procedure, algorithms are programmed to auto detect implausible value while entering data, and to prompt message for incomplete entry. Ad hoc discussions will be organized with study leads to clarify actions required to resolve data issues.

## ETHICS AND DISSEMINATION

The study has been approved by the Centralised Institutional Review Board of SingHealth (2021/2247) and registered at ClinicalTrials.gov (NCT05207059). Written informed consent will be obtained from all participants (Supplemental material). The findings will be published in peer-reviewed journals and disseminated to national and international policy makers.

For peer review only

**DISCUSSION**

The existing MOC in maternal-child health in Singapore and in many developed countries emphasizes disease treatment with less emphasis on health promotion. Most women are not prepared for pregnancy, and many have poor metabolic and mental health. This lack of preparedness carries over in the postpartum phase after the child is born. A woman's own needs are often neglected in the immediate postpartum phase, when the attention is primarily focused on caring for the newborn. Coupled with unexpected physical and emotional challenges in this new phase of life, it culminates in a cycle of poor metabolic and mental health. Women living with overweight and obesity represent a particularly high-risk group for poor metabolic and mental health.[49 50] The current obesity management strategy has three fundamental gaps. Firstly, the timing of intervention is too far downstream in adulthood. Secondly, there is weak foundational knowledge of nutrition and physical activity, with poor awareness and insight about metabolic health. Thirdly, there is no structured and coordinated care plan for these women while planning to conceive, which would be an ideal window for intervention during the crucial period of gametogenesis.[51] To address these challenges, there is a need to invert the pyramid of care and focus our effects upstream, starting from preconception.

HELMS aims to address the twin challenges of metabolic and mental health disorders through a series of interventions, with the goal of providing the best and most equal start to life for children. Through its life-course approach, HELMS recognises the additive effects of influences on a person's life that will impact the future health trajectory. It aims to impart new mental models of nutrition, physicality, and mental wellness, by guiding, supporting, and empowering women through an integrated journey from preconception to postpartum, to address metabolic and mental health challenges. We hope this unique holistic approach with

For peer review only

a continuum of care across the early life-course, starting from preconception, pregnancy until the postpartum period will build a strong foundation for healthy women and secure a healthy start for future generations.

Drawing on the experience and evidence derived from two local prospective observational mother-offspring cohorts conducted at KKH and National University Hospital (NUH) since 2008 – Growing Up in Singapore Toward Healthy Outcomes (GUSTO) and the S-PRESTO),[45 52] HELMS seek to examine the impact of integrated interventions, initiated preconceptionally, on metabolic and mental health of women living with overweight or obesity. Extensive biosampling and detailed phenotyping of mother, father, and child will allow longitudinal assessment of changes in health behaviours including diet, physical activity and sleep, maternal metabolic profile, and depression risk. The biosamples will provide an important platform for biomarker discovery, validate integrated HELMS interventions, and pave the way for new guidelines for lifestyle programs from the preconception to postpartum stages.

Despite the strong evidence base for adverse maternal and child outcomes in overweight and obese women, as well as the benefit of lifestyle interventions in the preconception and pregnancy phases, integrated HELMS interventions are novel and have not established unequivocal evidence of benefit. Thus, HELMS is conceptualized as a single arm intervention trial in women living with overweight or obesity to obtain preliminary evidence of efficacy of intervention. A subsequent larger pragmatic trial is envisaged to validate the efficacy of the intervention. The main limitations of this study design include the inability to distinguish between the effect of intervention, a placebo effect, and the effect of natural history, including regression to the mean. Given the similarity in study design to the

For peer review only



For peer review only - http://bmjopen.bmj.com/site/about/guidelines.xhtml

prospective observational cohort S-PRESTO, with similar time points of follow-up and biosampling, there is the potential for comparison with this historical cohort. HELMS is a single-centre study of the Asian population, involving only English-speaking participants, thus, the external validity may be limited to this population, and caution will need to be exercised in generalizing results across different settings and populations. Finally, the interventions are tailored to the woman / mother, with only passive intervention for her partner. The environment of the woman plays an important role in her compliance to lifestyle interventions,[53] which may undermine the impact of our program. On the other hand, if the success of the HELMS interventions extends to that of her immediate family, we would also have limited insights with minimal measurements on the partner, to reduce the burden of involvement in the study.

The major strength of this study is the extensive biosampling and detailed phenotyping of mother, father, and child, which will allow longitudinal assessment of changes in health behaviours including diet, physical activity and sleep, maternal metabolic profile, and depression risk. This provides an important platform for biomarker discovery, validation of integrated HELMS interventions, and pave the way for new guidelines for lifestyle programs throughout the life-course. We acknowledge the main limitation of this single-arm trial, where we are unable to distinguish between the effect of intervention, a placebo effect, or the effect of natural history. In addition, HELMS is conducted in a developed country with only English-speaking Asian participants, since English is the language used in our assessment tools and advisories, which may limit its external validity and caution should be exercised before generalizing our findings to other settings and population.

**Trial Status**


For peer review only - http://bmjopen.bmj.com/site/about/guidelines.xhtml

HELMS recruitment commenced in April 2022 and is expected to be completed in June 2024. The current protocol is version 1, dated 16 Dec 2021.

## AUTHORS CONTRIBUTIONS

Conceptualization, J.Y.K.C, F.Y, C.W.K and S.L.L; methodology, J.Y.K.C, F.Y, K.M.G, C.W.K and S.L.L; writing—original draft preparation, C.W.K, S.L.L and Y.F; writing—review and editing, all authors.; funding acquisition, M.C.C, F.Y and J.K.Y.C. All authors have read and agreed to the published version of the manuscript.

## FUNDING STATEMENT

This research was supported by the KKH Health Services Model of Care Transformation Fund (MoCTF) Grant (MoCTF/01/2020, MoCTF/02/2020 and MoCTF/03/2020) and the Lien Foundation Optimising Maternal and Child Health Programme Fund (Grant number not applicable). The funding body did not influence either the data collection and analysis or the writing and the decision to submit the manuscript. C.W.K. and J.K.Y.C. are supported by the National Medical Research Council, Ministry of Health, Singapore (NMRC/MOH-000596-00 and NMRC/CSA-SI-008-2016, respectively). K.M.G. is supported by the National Institute for Health Research (NIHR Senior Investigator (NF-SI-0515-10042), NIHR Southampton 1000DaysPlus Global Nutrition Research Group (17/63/154) and NIHR Southampton Biomedical Research Center (IS-BRC-1215-20004), British Heart Foundation (RG/15/17/3174) and the European Union (Erasmus+ Programme ImpENSA 598488-EPP-1-2018-1-DE-EPPKA2-CBHE-JP).

## COMPETING INTERESTS STATEMENT

K.M.G. and F.Y. received reimbursement for speaking at conferences sponsored by companies that sell nutritional products. K.M.G. is part of an academic consortium that received research funding from Abbott Nutrition, Nestle and Danone. All other authors declare no competing interests.

**ACKNOWLEDGEMENTS**

The authors would like to thank KK Women's and Children's Hospital for the institutional support received during this study. We would also like our multi-disciplinary team of collaborators including, but not limited to, Dr Helen Chen, Dr Han Wee Meng, Dr Mary Chong, Dr Jacinth Tan, Dr Saumya Jamuar, Dr Salvatore Albani, Dr Joanne Yoong, Dr Ng Wei Yen, Dr Jeremy Lim, Dr Michael Chee, and Dr Falk Mueller-Riemen-Schneider. The authors also thank the contributions of Ms Ng Xiang Wen, Ms Lai Lan Tian, Ms Rachael Loo Si Xuan, and Ms Faith Liew Hui Hua for their administrative support.

For peer review only



For peer review only - http://bmjopen.bmj.com/site/about/guidelines.xhtml

## REFERENCES

1. GBD 2015 Risk Factors Collaborators. Global, regional, and national comparative risk assessment of 79 behavioural, environmental and occupational, and metabolic risks or clusters of risks, 1990–2015: a systematic analysis for the Global Burden of Disease Study 2015. *Lancet* 2016;388:1659–1724.
2. World Health Organization. *Depression*. 2021. https://www.who.int/news-room/fact-sheets/detail/depression. [Accessed 29 Sep 2021].
3. Kim SR, Kim HN, Song SW. Associations Between Mental Health, Quality of Life, and Obesity/Metabolic Risk Phenotypes. *Metab Syndr Relat Disord* 2020;18:347–52.
4. Limon VM, Lee M, Gonzalez B, *et al*. The impact of metabolic syndrome on mental health-related quality of life and depressive symptoms. *Qual Life Res* 2020;29:2063–72.
5. Poston L, Caleyachetty R, Cnattingius S, *et al*. Preconceptional and maternal obesity: epidemiology and health consequences. *Lancet Diabetes Endocrinol* 2016;4:1025–36.
6. Becker M, Weinberger T, Chandy A, *et al*. Depression During Pregnancy and Postpartum. *Curr Psychiatry Rep* 2016;18:32.
7. Dalrymple KV, El-Heis S, Godfrey KM. Maternal weight and gestational diabetes impacts on child health. *Current Opinion in Clinical Nutrition and Metabolic Care* – in press.
8. Loy SL, Cheung YB, Soh SE, *et al*. Female adiposity and time-to-pregnancy: a multiethnic prospective cohort. *Hum Reprod* 2018;33:2141–49.
9. Kee MZL, Ponmudi S, Phua DY, *et al*. Preconception origins of perinatal maternal mental health. *Arch Womens Ment Health* 2021;24:605–18.
10. Law EC, Aishworiya R, Cai S, *et al*. Income disparity in school readiness and the mediating role of perinatal maternal mental health: a longitudinal birth cohort study. *Epidemiol Psychiatr Sci* 2021;30:e6.

For peer review only

11. Poston L, Bell R, Croker H, *et al*. Effect of a behavioural intervention in obese pregnant women (the UPBEAT study): a multicentre, randomised controlled trial. *Lancet Diabetes Endocrinol* 2015;3:767–77.

12. Lavender TJ, Ebert L, Jones D. An evaluation of perinatal mental health interventions: An integrative literature review. *Women Birth* 2016;29:399–406.

13. Hurt L, Paranjothy S, Lucas PJ, *et al*. Interventions that enhance health services for parents and infants to improve child development and social and emotional well-being in high-income countries: a systematic review. *BMJ Open* 2018;8:e014899.

14. Thangaratinam S, Rogozinska E, Jolly K, *et al*. Effects of interventions in pregnancy on maternal weight and obstetric outcomes: meta-analysis of randomised evidence. *BMJ* 2012;344:1–15.

15. Rogozińska E, Chamillard M, Hitman GA, *et al*. Nutritional manipulation for the primary prevention of gestational diabetes mellitus: a meta-analysis of randomised studies. *PloS One* 2015;10:1–21.

16. Dodd JM, Cramp C, Sui Z, *et al*. The effects of antenatal dietary and lifestyle advice for women who are overweight or obese on maternal diet and physical activity: the LIMIT randomised trial. *BMC Med* 2014;12:161.

17. Simmons D, Devlieger R, van Assche A, *et al*. Effect of Physical Activity and/or Healthy Eating on GDM Risk: The DALI Lifestyle Study. *J Clin Endocrinol Metab* 2017;102:903–13.

18. Okesene-Gafa KAM, Li M, McKinlay CJD, *et al*. Effect of antenatal dietary interventions in maternal obesity on pregnancy weight-gain and birthweight: Healthy Mums and Babies (HUMBA) randomized trial. *Am J Obstet Gynecol* 2019;221:152.e1–152.e13.

For peer review only



For peer review only - http://bmjopen.bmj.com/site/about/guidelines.xhtml

19. Hanson M, Barker M, Dodd JM, *et al*. Interventions to prevent maternal obesity before conception, during pregnancy, and post partum. *Lancet Diabetes Endocrinol* 2017;5:65–76.

20. Hanson MA, Gluckman PD. Developmental origins of health and disease: moving from biological concepts to interventions and policy. *Int J Gynaecol Obstet* 2011;115:S3–5.

21. Stephenson J, Schoenaker D, Hinton W, Poston L, Barker M, Alwan N, Godfrey K, Hanson M, de Lusignan S. A 'wake-up call' for preconception health: a clinical review. *Br J General Practice* 2021; 71:233-236.

22. Barker M, Dombrowski SU, Colbourn T, *et al*. Intervention strategies to improve nutrition and health behaviours before conception. *Lancet* 2018;391:1853–64.

23. Lan L, Harrison CL, Misso M, *et al*. Systematic review and meta-analysis of the impact of preconception lifestyle interventions on fertility, obstetric, fetal, anthropometric and metabolic outcomes in men and women. *Hum Reprod* 2017;32:1925–40.

24. Jacob CM, Newell M-L, Hanson M. Narrative review of reviews of preconception interventions to prevent an increased risk of obesity and non-communicable diseases in children. *Obes Rev* 2019;20:5–17.

25. Wekker V, Huvinen E, van Dammen L, *et al*. Long-term effects of a preconception lifestyle intervention on cardiometabolic health of overweight and obese women. *Eur J Public Health* 2019;29:308–14.

26. Hussein N, Kai J, Qureshi N. The effects of preconception interventions on improving reproductive health and pregnancy outcomes in primary care: A systematic review. *Eur J Gen Pract* 2016;22:42–52.

27. Bütikofer A, Løken KV, Salvanes KG. Infant health care and long-term outcomes. *The Review of Economics and Statistics* 2019;101:341–54.



For peer review only - http://bmjopen.bmj.com/site/about/guidelines.xhtml

28. Hanson M, Gluckman P. Developmental origins of noncommunicable disease: population and public health implications. *Am J Clin Nutr* 2011;94:1754S–1758S.

29. Ku CW, Leow SH, Ong LS, *et al*. Development of a Lifestyle Intervention Program for Overweight or Obese Preconception, Pregnant and Postnatal Women: a Qualitative Study. PREPRINT (version 1) *Research Square* 2021. https://doi.org/10.21203/rs.3.rs-877807/v1

30. Chee Wai Ku, Rachael Si Xuan Loo, Cheryl Jia En Lim, *et al*. Development and Validation of a Lifestyle Behavior Tool in Overweight and Obese Women through Qualitative and Quantitative Approaches. *Nutrients* 2021, 13, 4553.

31. Ajzen I. The theory of planned behavior. *Organizational Behavior and Human Decision Processes* 1991;50:179–211.

32. Thaler RH, Sunstein CR. *Nudge: improving decisions about health. Wealth, and Happiness*. Yale University Press, 2008.

33. Shilts MK, Horowitz M, Townsend MS. Goal setting as a strategy for dietary and physical activity behavior change: a review of the literature. *Am J Health Promot* 2004;19:81–93.

34. Armitage CJ, Conner M. Efficacy of the Theory of Planned Behaviour: a meta-analytic review. *Br J Soc Psychol* 2001;40:471–99.

35. Cox JL, Holden JM, Sagovsky R. Detection of postnatal depression. Development of the 10-item Edinburgh Postnatal Depression Scale. *Br J Psychiatry* 1987;150:782–86.

36. Rosen R, Brown C, Heiman J, *et al*. The Female Sexual Function Index (FSFI): a multidimensional self-report instrument for the assessment of female sexual function. *J Sex Marital Ther* 2000;26:191–208.

For peer review only



For peer review only - http://bmjopen.bmj.com/site/about/guidelines.xhtml

37. Lim SX, Colega MT, M Ayob MN, *et al*. Identification and reproducibility of dietary patterns assessed with a FFQ among women planning pregnancy. *Public Health Nutr* 2021;24:2437–46.

38. Stunkard AJ, Messick S. The three-factor eating questionnaire to measure dietary restraint, disinhibition and hunger. *J Psychosom Res* 1985;29:71–83.

39. Craig CL, Marshall AL, Sjöström M, *et al*. International physical activity questionnaire: 12-country reliability and validity. *Med Sci Sports Exerc* 2003;35:1381–95.

40. IPAQ research committee. Guidelines for data processing and analysis of the international physical activity questionnaire-short and long forms, 2004.

41. Padmapriya N, Shen L, Soh SE, *et al*. Physical Activity and Sedentary Behavior Patterns Before and During Pregnancy in a Multi-ethnic Sample of Asian Women in Singapore. *Matern Child Health J* 2015;19:2523–35.

42. Buysse DJ, Reynolds CF, Monk TH, *et al*. The Pittsburgh Sleep Quality Index: a new instrument for psychiatric practice and research. *Psychiatry Res* 1989;28:193–213.

43. Spira A. The use of fecundability in epidemiological surveys. *Hum Reprod* 1998;13:1753–6.

44. Wilcox AJ. *Fertility and pregnancy: an epidemiologic perspective*. United Kingdom: Oxford University Press, 2010.

45. Loo EXL, Soh SE, Loy S, *et al*. Cohort profile: Singapore Preconception Study of Long-Term Maternal and Child Outcomes (S-PRESTO). *Eur J Epidemiol* 2021;36:129–42.

46. Royston P. Multiple Imputation of Missing Values. *Stata J* 2004;4:227–41.

47. Cheung YB. Analysis of repeated measurements and clustered data. In: *Statistical analysis of human growth and development*. USA, Boca Raton (FL): CRC Press, 2014.

48. Rubin DB. *Multiple imputation for nonresponse in surveys*. USA, New York: John Wiley & Sons, 2004.

For peer review only



For peer review only - http://bmjopen.bmj.com/site/about/guidelines.xhtml

49. Marchi J, Berg M, Dencker A, *et al*. Risks associated with obesity in pregnancy, for the mother and baby: a systematic review of reviews. *Obes Rev* 2015;16:621-38.

50. Dachew BA, Ayano G, Betts K, *et al*. The impact of pre-pregnancy BMI on maternal depressive and anxiety symptoms during pregnancy and the postpartum period: A systematic review and meta-analysis. *J Affect Disord* 2021;281:321-30.

51. Catalano PM, Shankar K. Obesity and pregnancy: mechanisms of short term and long term adverse consequences for mother and child. *BMJ* 2017;356:j1.

52. Soh SE, Tint MT, Gluckman PD, *et al*. Cohort profile: Growing Up in Singapore Towards healthy Outcomes (GUSTO) birth cohort study. *Int J Epidemiol* 2014;43:1401–09.

53. Burgess E, Hassmén P, Pumpa KL. Determinants of adherence to lifestyle intervention in adults with obesity: a systematic review. *Clin Obes* 2017;7:123-35.

For peer review only

## FIGURES

**Figure 1**. The Healthy Early Life Moments in Singapore (HELMS) framework, comprising preconception, pregnancy, and postpartum dyad care. Through maternal behavioural intervention, the overarching aim is to improve metabolic and mental health in women living with obesity, and to improve early child growth. Specifically, HELMS aims to improve women's fertility, obstetric outcomes, physical and mental recovery from birth, feeding habits and growth of the child.

**Figure 2.** HELMS study flow diagram. Preconception visits are coloured green, pregnancy visits pink, and postpartum visits blue. HELMS, Healthy Early Life Moments in Singapore.

For peer review only

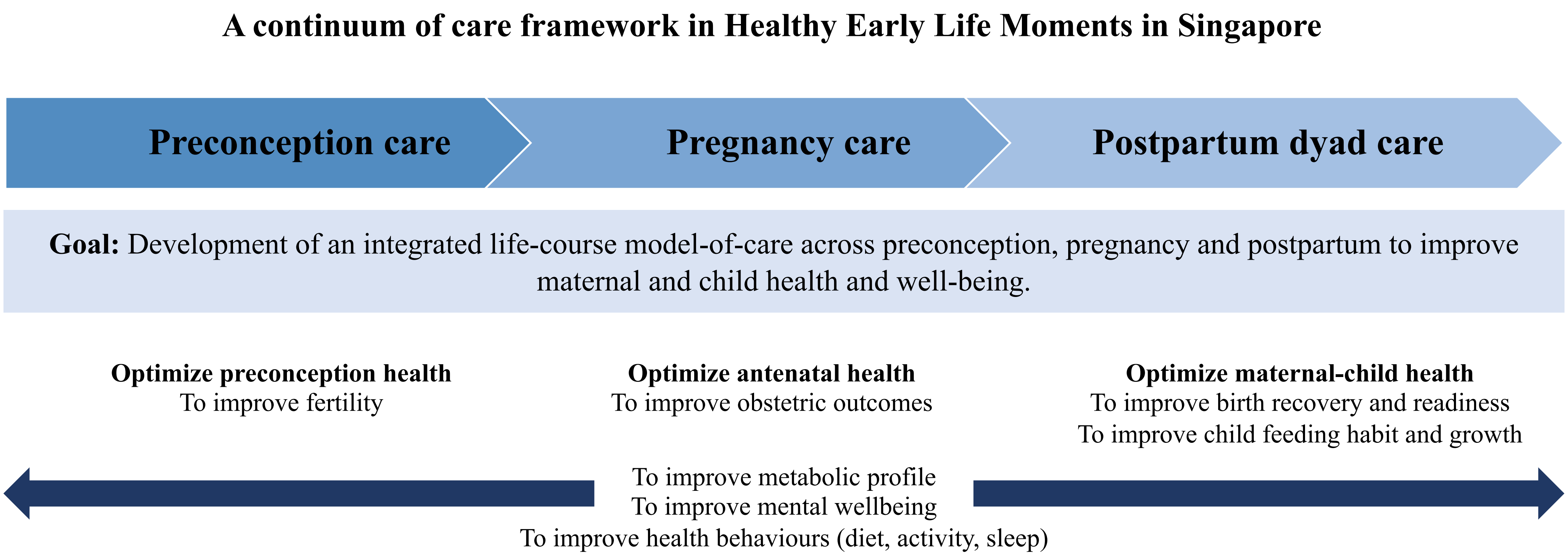
A continuum of care framework in Healthy Early Life Moments in Singapore
Preconception care
Pregnancy care
Postpartum dyad care
Goal: Development of an integrated life-course model-of-care across preconception, pregnancy and postpartum to improve maternal and child health and well-being.
Optimize preconception health
To improve fertility
Optimize antenatal health
To improve obstetric outcomes
Optimize maternal-child health
To improve birth recovery and readiness
To improve child feeding habit and growth
To improve metabolic profile
To improve mental wellbeing
To improve health behaviours (diet, activity, sleep)

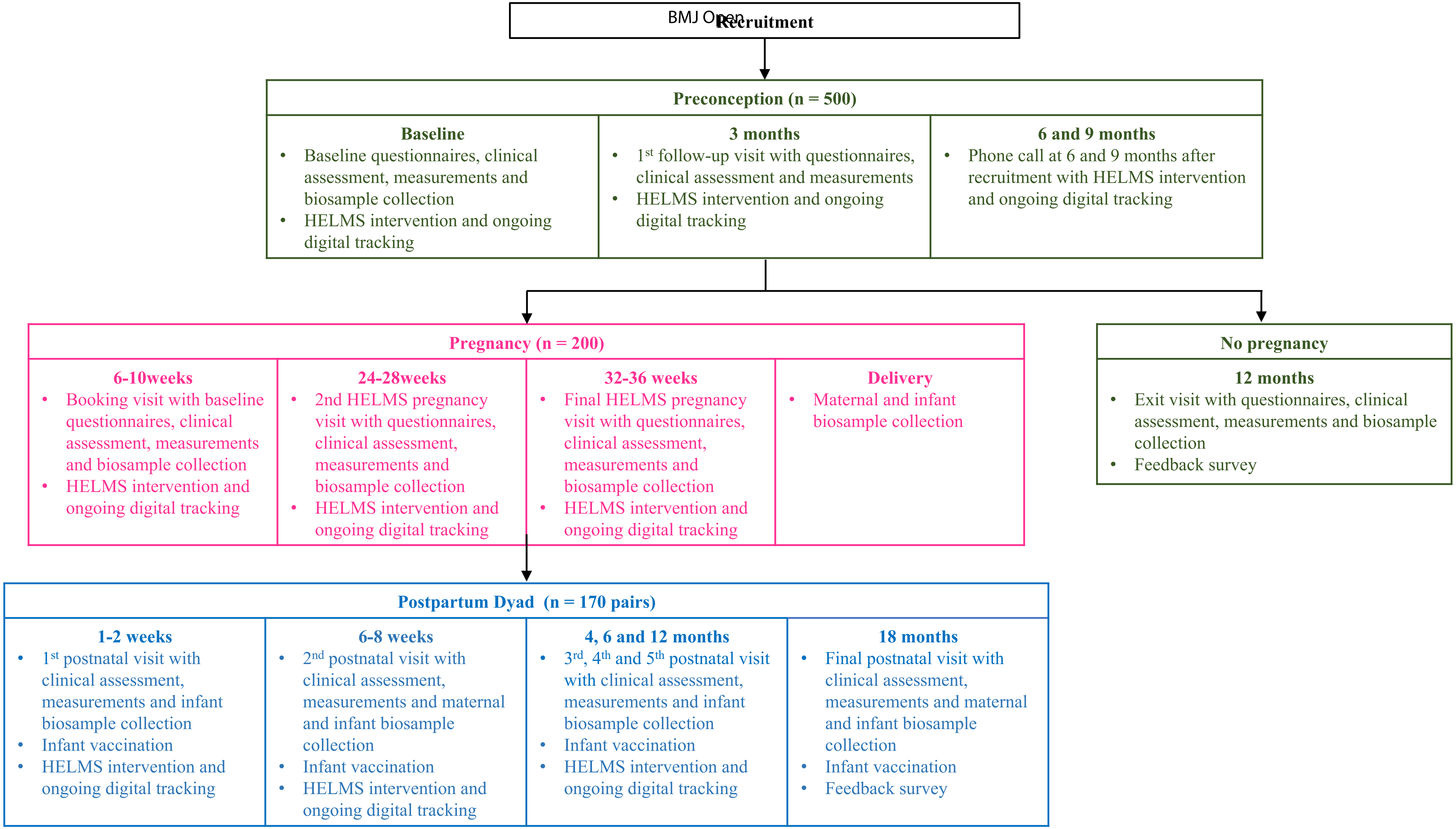
Recruitment
Preconception (n = 500)
Baseline
• Baseline questionnaires, clinical assessment, measurements and biosample collection
• HELMS intervention and ongoing digital tracking
3 months
• 1st follow-up visit with questionnaires, clinical assessment and measurements
• HELMS intervention and ongoing digital tracking
6 and 9 months
• Phone call at 6 and 9 months after recruitment with HELMS intervention and ongoing digital tracking
Pregnancy (n = 200)
6-10weeks
• Booking visit with baseline questionnaires, clinical assessment, measurements and biosample collection
• HELMS intervention and ongoing digital tracking
24-28weeks
• 2nd HELMS pregnancy visit with questionnaires, clinical assessment, measurements and biosample collection
• HELMS intervention and ongoing digital tracking
32-36 weeks
• Final HELMS pregnancy visit with questionnaires, clinical assessment, measurements and biosample collection
• HELMS intervention and ongoing digital tracking
Delivery
• Maternal and infant biosample collection
No pregnancy
12 months
• Exit visit with questionnaires, clinical assessment, measurements and biosample collection
• Feedback survey
Postpartum Dyad (n = 170 pairs)
1-2 weeks
• 1st postnatal visit with clinical assessment, measurements and infant biosample collection
• Infant vaccination
• HELMS intervention and ongoing digital tracking
6-8 weeks
• 2nd postnatal visit with clinical assessment, measurements and maternal and infant biosample collection
• Infant vaccination
• HELMS intervention and ongoing digital tracking
4, 6 and 12 months
• 3rd, 4th and 5th postnatal visit with clinical assessment, measurements and infant biosample collection
• Infant vaccination
• HELMS intervention and ongoing digital tracking
18 months
• Final postnatal visit with clinical assessment, measurements and maternal and infant biosample collection
• Infant vaccination
• Feedback survey

SPIRIT

STANDARD PROTOCOL ITEMS: RECOMMENDATIONS FOR INTERVENTIONAL TRIALS

SPIRIT 2013 Checklist: Recommended items to address in a clinical trial protocol and related documents*

| Section/item | Item No | Description | Addressed on page number |
|---|---|---|---|
| **Administrative information** | | | |
| Title | 1 | Descriptive title identifying the study design, population, interventions, and, if applicable, trial acronym | ______1_______ |
| Trial registration | 2a | Trial identifier and registry name. If not yet registered, name of intended registry | ______4______ |
| | 2b | All items from the World Health Organization Trial Registration Data Set | ______4_______ |
| Protocol version | 3 | Date and version identifier | ______23______ |
| Funding | 4 | Sources and types of financial, material, and other support | ______24_____ |
| Roles and responsibilities | 5a | Names, affiliations, and roles of protocol contributors | ______23_____ |
| | 5b | Name and contact information for the trial sponsor | ______24_____ |
| | 5c | Role of study sponsor and funders, if any, in study design; collection, management, analysis, and interpretation of data; writing of the report; and the decision to submit the report for publication, including whether they will have ultimate authority over any of these activities | ______23_____ |
| | 5d | Composition, roles, and responsibilities of the coordinating centre, steering committee, endpoint adjudication committee, data management team, and other individuals or groups overseeing the trial, if applicable (see Item 21a for data monitoring committee) | ______22_______ |

For peer review only



For peer review only - http://bmjopen.bmj.com/site/about/guidelines.xhtml

**Introduction**

| | | | |
|---|---|---|---|
| Background and rationale | 6a | Description of research question and justification for undertaking the trial, including summary of relevant studies (published and unpublished) examining benefits and harms for each intervention | ______5-7____ |
| | 6b | Explanation for choice of comparators | ______5-7____ |
| Objectives | 7 | Specific objectives or hypotheses | ______7_______ |
| Trial design | 8 | Description of trial design including type of trial (eg, parallel group, crossover, factorial, single group), allocation ratio, and framework (eg, superiority, equivalence, noninferiority, exploratory) | ______8-9_____ |

**Methods: Participants, interventions, and outcomes**

| | | | |
|---|---|---|---|
| Study setting | 9 | Description of study settings (eg, community clinic, academic hospital) and list of countries where data will be collected. Reference to where list of study sites can be obtained | ______8-9_____ |
| Eligibility criteria | 10 | Inclusion and exclusion criteria for participants. If applicable, eligibility criteria for study centres and individuals who will perform the interventions (eg, surgeons, psychotherapists) | ______10_____ |
| Interventions | 11a | Interventions for each group with sufficient detail to allow replication, including how and when they will be administered | ______11-14__ |
| | 11b | Criteria for discontinuing or modifying allocated interventions for a given trial participant (eg, drug dose change in response to harms, participant request, or improving/worsening disease) | ______10______ |
| | 11c | Strategies to improve adherence to intervention protocols, and any procedures for monitoring adherence (eg, drug tablet return, laboratory tests) | ______15_______ |
| | 11d | Relevant concomitant care and interventions that are permitted or prohibited during the trial | ______11-14____ |
| Outcomes | 12 | Primary, secondary, and other outcomes, including the specific measurement variable (eg, systolic blood pressure), analysis metric (eg, change from baseline, final value, time to event), method of aggregation (eg, median, proportion), and time point for each outcome. Explanation of the clinical relevance of chosen efficacy and harm outcomes is strongly recommended | ______15-17____ |
| Participant timeline | 13 | Time schedule of enrolment, interventions (including any run-ins and washouts), assessments, and visits for participants. A schematic diagram is highly recommended (see Figure) | _____Figure 2__ |



For peer review only - http://bmjopen.bmj.com/site/about/guidelines.xhtml

| | | | |
|---|---|---|---|
| Sample size | 14 | Estimated number of participants needed to achieve study objectives and how it was determined, including clinical and statistical assumptions supporting any sample size calculations | _____19________ |
| Recruitment | 15 | Strategies for achieving adequate participant enrolment to reach target sample size | _____10________ |

**Methods: Assignment of interventions (for controlled trials)**

Allocation:

| | | | |
|---|---|---|---|
| Sequence generation | 16a | Method of generating the allocation sequence (eg, computer-generated random numbers), and list of any factors for stratification. To reduce predictability of a random sequence, details of any planned restriction (eg, blocking) should be provided in a separate document that is unavailable to those who enrol participants or assign interventions | ______NA______ |
| Allocation concealment mechanism | 16b | Mechanism of implementing the allocation sequence (eg, central telephone; sequentially numbered, opaque, sealed envelopes), describing any steps to conceal the sequence until interventions are assigned | ______NA______ |
| Implementation | 16c | Who will generate the allocation sequence, who will enrol participants, and who will assign participants to interventions | ______NA______ |
| Blinding (masking) | 17a | Who will be blinded after assignment to interventions (eg, trial participants, care providers, outcome assessors, data analysts), and how | ______NA______ |
| | 17b | If blinded, circumstances under which unblinding is permissible, and procedure for revealing a participant's allocated intervention during the trial | ______NA______ |

**Methods: Data collection, management, and analysis**

| | | | |
|---|---|---|---|
| Data collection methods | 18a | Plans for assessment and collection of outcome, baseline, and other trial data, including any related processes to promote data quality (eg, duplicate measurements, training of assessors) and a description of study instruments (eg, questionnaires, laboratory tests) along with their reliability and validity, if known. Reference to where data collection forms can be found, if not in the protocol | _____15-17_____ |
| | 18b | Plans to promote participant retention and complete follow-up, including list of any outcome data to be collected for participants who discontinue or deviate from intervention protocols | ______15-17___ |

| | | | |
|---|---|---|---|
| Data management | 19 | Plans for data entry, coding, security, and storage, including any related processes to promote data quality (eg, double data entry; range checks for data values). Reference to where details of data management procedures can be found, if not in the protocol | _____22-23____ |
| Statistical methods | 20a | Statistical methods for analysing primary and secondary outcomes. Reference to where other details of the statistical analysis plan can be found, if not in the protocol | ____18_____ |
| | 20b | Methods for any additional analyses (eg, subgroup and adjusted analyses) | ____18____ |
| | 20c | Definition of analysis population relating to protocol non-adherence (eg, as randomised analysis), and any statistical methods to handle missing data (eg, multiple imputation) | _____18____ |
| **Methods: Monitoring** | | | |
| Data monitoring | 21a | Composition of data monitoring committee (DMC); summary of its role and reporting structure; statement of whether it is independent from the sponsor and competing interests; and reference to where further details about its charter can be found, if not in the protocol. Alternatively, an explanation of why a DMC is not needed | _____23________ |
| | 21b | Description of any interim analyses and stopping guidelines, including who will have access to these interim results and make the final decision to terminate the trial | ______NA______ |
| Harms | 22 | Plans for collecting, assessing, reporting, and managing solicited and spontaneously reported adverse events and other unintended effects of trial interventions or trial conduct | ______NA______ |
| Auditing | 23 | Frequency and procedures for auditing trial conduct, if any, and whether the process will be independent from investigators and the sponsor | ______22_______ |
| **Ethics and dissemination** | | | |
| Research ethics approval | 24 | Plans for seeking research ethics committee/institutional review board (REC/IRB) approval | _____22______ |
| Protocol amendments | 25 | Plans for communicating important protocol modifications (eg, changes to eligibility criteria, outcomes, analyses) to relevant parties (eg, investigators, REC/IRBs, trial participants, trial registries, journals, regulators) | _____NA_______ |

| | | | |
|---|---|---|---|
| Consent or assent | 26a | Who will obtain informed consent or assent from potential trial participants or authorised surrogates, and how (see Item 32) | ______8, 22______ |
| | 26b | Additional consent provisions for collection and use of participant data and biological specimens in ancillary studies, if applicable | _______NA______ |
| Confidentiality | 27 | How personal information about potential and enrolled participants will be collected, shared, and maintained in order to protect confidentiality before, during, and after the trial | _______23________ |
| Declaration of interests | 28 | Financial and other competing interests for principal investigators for the overall trial and each study site | _______24________ |
| Access to data | 29 | Statement of who will have access to the final trial dataset, and disclosure of contractual agreements that limit such access for investigators | ______NA________ |
| Ancillary and post-trial care | 30 | Provisions, if any, for ancillary and post-trial care, and for compensation to those who suffer harm from trial participation | _______NA_______ |
| Dissemination policy | 31a | Plans for investigators and sponsor to communicate trial results to participants, healthcare professionals, the public, and other relevant groups (eg, via publication, reporting in results databases, or other data sharing arrangements), including any publication restrictions | _______NA_______ |
| | 31b | Authorship eligibility guidelines and any intended use of professional writers | ______NA________ |
| | 31c | Plans, if any, for granting public access to the full protocol, participant-level dataset, and statistical code | ______NA________ |
| **Appendices** | | | |
| Informed consent materials | 32 | Model consent form and other related documentation given to participants and authorised surrogates | ______NA________ |
| Biological specimens | 33 | Plans for collection, laboratory evaluation, and storage of biological specimens for genetic or molecular analysis in the current trial and for future use in ancillary studies, if applicable | ______17_________ |

*It is strongly recommended that this checklist be read in conjunction with the SPIRIT 2013 Explanation & Elaboration for important clarification on the items. Amendments to the protocol should be tracked and dated. The SPIRIT checklist is copyrighted by the SPIRIT Group under the Creative Commons "Attribution-NonCommercial-NoDerivs 3.0 Unported" license.



For peer review only - http://bmjopen.bmj.com/site/about/guidelines.xhtml
